# The effect of exercise interventions on reducing the risk of depressive and cognitive disorders in post-stroke—a systematic review and meta-analysis

**DOI:** 10.3389/fneur.2025.1564347

**Published:** 2025-03-24

**Authors:** Zixian Yang, Shaokun Qin, Jiaxing Li, Cong Li, Ye Lu, Pei He, Jia Liu, Lin Pei

**Affiliations:** ^1^School of Basic Medicine, Hebei University of Chinese Medicine, Shijiazhuang, Hebei, China; ^2^Section of Science and Education, The Second Affiliated Hospital of Hebei University of Chinese Medicine, Dingzhou, Hebei, China; ^3^Shijiazhuang Medical College, Shijiazhuang, Hebei, China; ^4^Key Research Laboratory of Phlegm Stagnation Syndrome and Treatment in Hebei Province, Hebei Academy of Chinese Medicine Sciences, Shijiazhuang, China; ^5^The Fourth Affiliated Hospital of Hebei University of Chinese Medicine, Shijiazhuang, Hebei, China

**Keywords:** stroke, cognitive, depressive, exercise, meta-analysis

## Abstract

**Background:**

Stroke patients often experience sequelae such as depressive symptoms, cognitive impairment, and abnormal physical function. Exercise intervention may be an effective and safe non-drug treatment to address these health issues.

**Objective:**

The aim of this meta-analytical review was to explore the effects of exercise intervention programs on depressive symptoms, cognitive function, physical function, and quality of life in stroke patients, as well as to identify appropriate exercise programs.

**Methods:**

Seven databases were searched from the library’s construction until 30 August 2024. A meta-analysis was performed, and the risk of bias was assessed using Review Manager 5.4. Sensitivity analysis was conducted using Stata 16.0 software, and the overall certainty of the evidence was rated using Grading of Recommendations, Assessment, Development, and Evaluation (GRADE) methods.

**Results:**

A total of 11,607 studies were identified. Among these, 20 studies, which included 1,848 patients, were considered eligible for this network meta-analysis. Compared to the control group, exercise significantly improved cognitive function (standard mean difference [SMD] = 1.08, 95% confidence interval [CI] = 0.40–1.75, *p* = 0.002), physical balance ability (mean difference [MD] = 0.80, 95% CI = 0.23–1.37, *p* < 0.01), physical walking ability (MD = 48.39, 95% CI = 8.06–88.72, *p* = 0.02), and quality of life. However, exercise had no significant effect on depressive symptoms (SMD = −0.2, 95% CI = −0.46–0.05, *p* = 0.11). A subgroup analysis indicated that a longer duration of exercise (> 3 months) can effectively improve depressive symptoms in stroke patients.

**Conclusion:**

The results indicated that cognitive function, balance, walking speed, and quality of life of stroke patients improved following exercise intervention, and longer exercise duration (> 3 months) contributed to alleviating the depressive symptoms of stroke patients. Therefore, we recommend that stroke patients engage in physical exercise 3 times a week for 1 h each session. The exercise duration should continue for at least 3 months to ensure the best therapeutic effect. Furthermore, determining exercise intensity should be a personalized process—carefully customized to align with the physical capabilities and limitations of each patient.

**Systematic review registration:**

https://www.crd.york.ac.uk/prospero, CRD42024520778.

## 1 Introduction

Stroke is the leading cause of acquired disability among adults worldwide (1), and stroke survivors are likely to experience long-term neurological complications (2). Stroke patients are more likely to develop depressive symptoms, cognitive impairment, and physical movement disorder after surgery; these complications adversely affect the quality of life, survival rates, and functional recovery of stroke patients (3–5).

One of the most prevalent long-term effects of stroke is post-stroke depression (PSD), which affects 11–41% of stroke survivors worldwide and is associated with a markedly higher risk of death. According to the depression scale, approximately 50% of stroke patients have PSD (6, 7). Up to one-third of stroke survivors may experience the severe consequences of cognitive impairment, which frequently follows a stroke (8). Research shows that stroke survivors with mild cognitive impairment face a twofold increased risk of death (9). Hemiplegia affects over 85% of stroke patients, leading to impaired upper limb function and decreased motor ability (10). This impairment significantly impacts balance and the extent of daily and social activities (11). National and international stroke treatment guidelines rarely emphasize the most effective clinical prevention and treatment strategies for stroke survivors (12). Currently, medication and psychotherapy are the standard treatments; however, these do not significantly enhance physical function and quality of life (13, 14).

There is a wealth of evidence supporting the protective role of exercise in cognitive and depressive disorders after stroke. Exercise intervention offers multiple benefits and effects that may enhance the cognitive function following a stroke (15), recovery of arm function, improvement of balance index and gait speed, and improvement of physical function and quality of life. Cognitive and depressive disorders have been shown to benefit from exercises (12, 16–18). Research shows that exercise can improve cardiovascular fitness, elevate blood levels of adrenaline and brain-derived neurotrophic factors, and positively supervise brain function, including growth factors, brain metabolism, neurotransmitters, oxygen availability, glucose regulation, and oxidative stress. These processes can enhance both depression and cognitive function (19). Although the efficacy of exercise in managing stroke sequelae is well established, research findings regarding the dose–response relationship of exercise in stroke patients remain inconsistent. Current literature offers limited guidance on the best exercise parameters for treating stroke-related impairments.

Therefore, the aim of this meta-analysis and systematic review is to thoroughly examine all published randomized controlled trials. The effects of post-stroke exercise on patients’ depression symptoms, cognitive function, physical function, and quality of life will be assessed to provide scientific support for future clinical practice and research.

## 2 Methods

### 2.1 Protocol and registration

This systematic review (No.: CRD42024520778) was registered with the Prospective Register of Systematic Reviews (PROSPERO) in April 2024. The Preferred Reporting Items for Systematic Reviews and Meta-Analyses (PRISMA) statement, along with a predefined methodology, were used for reporting this systematic review and meta-analyses.

### 2.2 Literature search

From the creation of the database until 30 August 2024, we conducted searches across seven databases: PubMed, Cochrane Library, Web of Science, Embase, Chinese National Knowledge Infrastructure (CNKI), Wanfang Data, and China Science and Technology Journal Database (VIPC). The medical subject words related to exercise, stroke, depression, cognitive impairment, and entry terms were retrieved, and the complete retrieval strategy is shown in Supplementary material.

### 2.3 Inclusion and exclusion criteria

The inclusion criteria were as follows: (1) study design: randomized controlled trials; (2) study population: patients with stroke; (3) stroke patients with a scale assessment of cognitive or depressive symptoms; (4) delivery of an exercise intervention of any modality; and (5) number of participants ≥30.

The exclusion criteria were as follows: (1) unstable medical history that could restrict participation (e.g., recent myocardial infarction); (2) simultaneously with other neurological disorders (e.g., amyotrophic lateral sclerosis, Parkinson’s disease, and multiple sclerosis); and (3) studies with missing information or abstracts for which, despite contacting the authors via email, the full text was not accessible.

### 2.4 Data collection

To eliminate duplicate records, all of the studies found through the literature search were imported into Endnote software (Clarivate Analytics). Two researchers independently vetted the literature using the inclusion and exclusion criteria.

The third reviewer resolved any disagreements by consensus or by consulting an expert. The following information was extracted: initial author, publication date, grouping technique, number of participants in each group, exercise and intervention mode, duration, outcome measures, and negative effects in both the experimental and the control groups.

### 2.5 Risk of bias assessment

Review Manager 5.4 software (Cochrane) was used to evaluate the quality of the included literature. A case-by-case assessment based on each included study included the following seven main items: (1) Random sequence generation (selection bias); (2) Allocation concealment (selection bias); (3) Blinding of participants and personnel (performance bias); (4) Blinding of outcome assessment (detection bias); (5) Incomplete outcome data (attrition bias); (6) Selective reporting (reporting bias); (7) Other biases; and (8) Other bias. Risk of bias was categorized as “high risk of bias (−)” “unclear (?)” “Or” low risk of bias (+).”

### 2.6 Strength of the evidence assessment

The quality of the evidence supporting the outcome was assessed using the Grading Assessment, Development, and Evaluation (GRADE) method of meta-analysis. Study limitations, inconsistent results, indirect outcomes, imprecise results, and publication bias were the five factors that could diminish the quality of evidence. The strength of evidence was categorized into four levels from high to low: strong, moderate, low, and very low. Since the intervention method is an exercise intervention, allocation masking and double-blinding could not be guaranteed, leading to a downgrade of all evidence by one level. If the forest plot crossed the equivalence line, or if the sample size of the included studies was too small, or the 95% confidence interval (CI) of the effect estimate was too wide, the evidence was downgraded by one level.

### 2.7 Statistical analysis

RevMan 5.4 was used for the evaluation of heterogeneity and merging data in this meta-analysis. Mean difference (MD) and standard mean difference (SMD) were used to represent continuous variables, whereas SMD was used to express continuous variables with distinct differences and units of measurement. *I*^2^ ≤ 50% was considered low heterogeneity, and the fixed effect model was used for the meta-analysis. Instead, a random-effects model was used for meta-analysis. In addition, subgroup analyses were performed with high statistical heterogeneity.

The sensitivity analyses were performed on a case-by-case basis; *p* < 0.05 was considered statistically significant according to the calculation of the 95% CI. The sensitivity analysis was conducted using Stata 16.0 software (StataCorp Limited Liability Company).

## 3 Results

**Figure 1:**
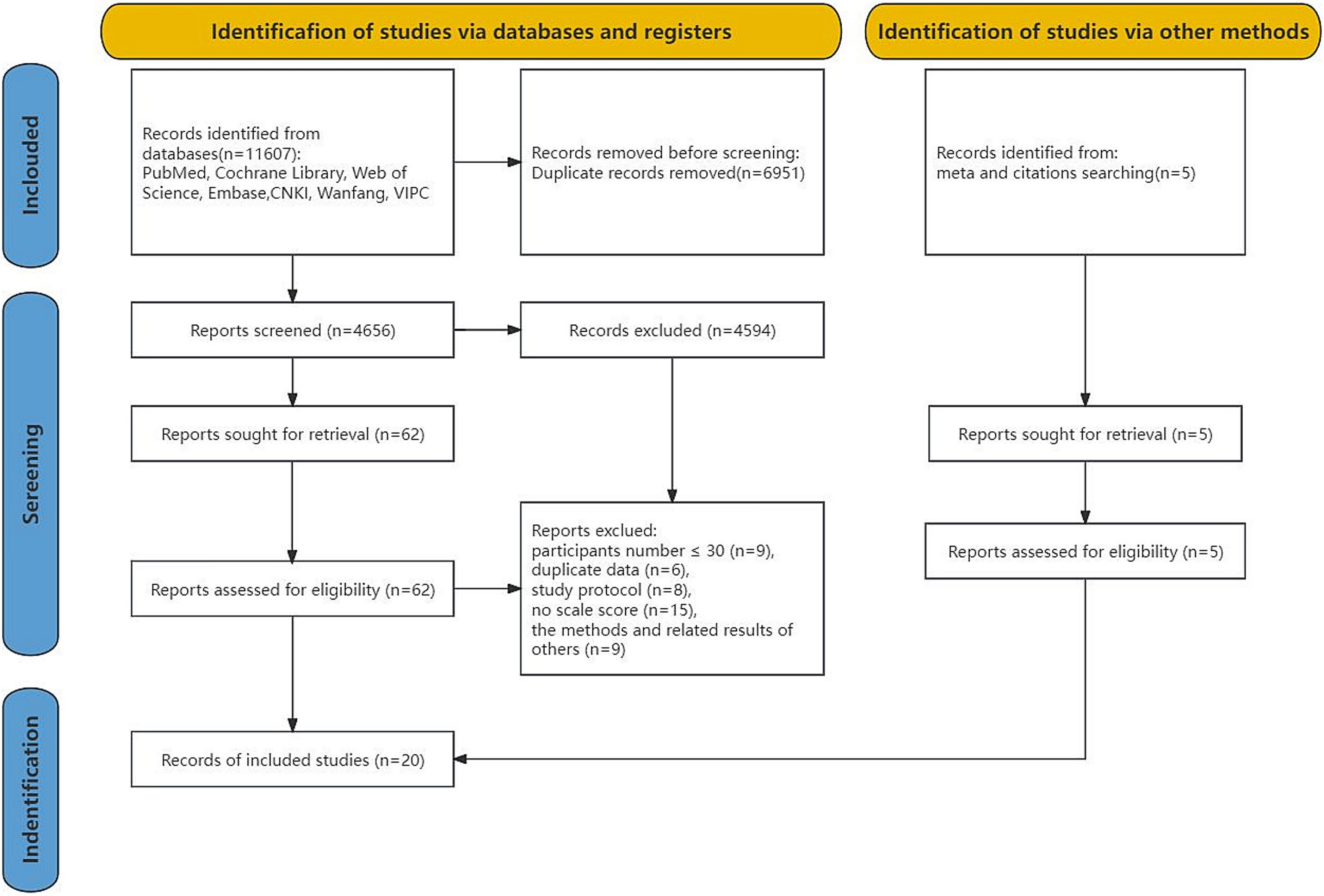
PRISMA flowchart.

After searching seven databases, a total of 11,607 studies were retrieved. After removing duplicates, 4,656 studies remained; their titles and abstracts were reviewed to determine if they met the inclusion criteria; as a result, 4,594 studies were excluded. The remaining 62 studies were independently reviewed by two authors (YZX and LJX). When a disagreement is difficult to resolve, the third author is often used as an arbiter to help reach a consensus on the issue. We manually searched relevant published meta-analyses, and the references of the included studies, of which five studies were available for inclusion. In total, 20 studies were included. The excluded cases were those with less than 30 participants (*n* = 9), duplicate date (*n* = 6), study protocol (*n* = 8), no scale score (*n* = 15), and other methods and related results (*n* = 9) (Figure 1).

### 3.1 Characteristics of the included studies

Characteristics of the included studies are shown in Table 1. This review only examined English-language literature and did not include studies published in other languages. The included studies, published between 2006 and 2023, enrolled 1,848 stroke patients with sample sizes ranging from 30 to 362. patients included experienced a wide range of time since their stroke, ranging from less than 1 month to more than 10 years. The 20 included studies from China (*n* = 3), the United Kingdom (*n* = 3), the United States (*n* = 3), the Netherlands (*n* = 2), Sweden (*n* = 2), Australia (*n* = 1), Canada (*n* = 1), Denmark (*n* = 1), Egypt (*n* = 1), Korea (*n* = 1), Norway (*n* = 1), Portugal (*n* = 1).

**Table 1:** Characteristics of the included studies.

Authors	Country	N(I/C)	Male	Female	Age	Mean age (I)	Mean age (C)	Stroke time
Deijle et al. (20)	Netherlands	60/59	70	49	≥18	64.7 ± 8.9	63.9 ± 10.6	<1 month
El-Tamawy et al. (21)	Egypt	15/15	21	9	48.4 ± 6.39	48.4 ± 6.39	49.67 ± 6.98	3–18 months
Gjellesvik et al. (22)	The United States	36/34	41	29	>18	57.6 ± 9.2	58.7 ± 9.2	3 months to 5 years
Harrington et al. (23)	The United Kingdom	124/119	132	111	NR	71 ± 10.5	70 ± 10.2	NR
Holmgren et al. (24)	Sweden	15/19	21	13	NR	77.7 ± 7.6	79.2 ± 7.5	3–6 months
Ihle-Hansen et al. (25)	Norway	177/185	219	143	>18	71.4 ± 11.3	72.0 ± 11.3	NR
Jiang et al. (26)	China	45/45	55	35	40–80	58.00 ± 3.13	58.11 ± 2.56	> 12 weeks
Koch et al. (27)	The United States	86/45	81	50	>18	59 ± 11	58 ± 12	<1 year
Krawcyk et al. (28)	Denmark	31/32	49	14	>18	63.7 ± 8.9	63.7 ± 9.2	<3 weeks
Lai et al. (3)	The United States	44/49	50	43	69.8 ± 10.3	70.4 ± 11.3	68.5 ± 9.0	NR
Lapointe et al. (29)	Canada	19/17	23	13	NR	71.8 ± 9.9	69.6 ± 10.7	>3 months
Maeneja et al. (19)	Portugal	17/17	19	15	≥40	55.12 ± 6.660	57.00 ± 10.23	NR
Mead et al. (30)	The United Kingdom	32/34	36	30	NR	71.7 ± 9.6	72.0 ± 10.4	NR
Moore et al. (31)	The United Kingdom	20/20	34	6	>50	68 ± 8	70 ± 11	>6 months
Sims et al. (32)	Australia	23/22	27	18	67.13 ± 15.23	67.95 ± 14.76	66.27 ± 16.01	13.2 months (SD 4.95)
Song et al. (33)	Korea	18/16	21	13	NR	58.72 ± 17.13	57.18 ± 10.65	NR
Vahlberg et al. (4)	Sweden	34/33	51	16	65–85	73.7 ± 5.3	72.6 ± 5.5	1–3 years
Zedlitz et al. (34)	Netherlands	38/45	43	40	18–70	54.8 ± 9.1	55.6 ± 8.8	≥4 months
Zhao et al. (35)	China	80/80	81	79	62.98 ± 12.85	62.21 ± 12.88	63.35 ± 12.90	1.5 months
Zheng et al. (36)	China	24/24	41	7	45–75	61.63 ± 9.21	62.75 ± 6.41	>3 months

### 3.2 Intervention characteristics

Regarding the types of exercise included in the intervention group, five studies were multicomponent exercises with three or more types (balance, cognitive, endurance, resistance, strength, and walking), three studies focused on aerobic exercises, three studies used combined aerobic and other exercises, three studies used traditional Chinese medicine exercises, two studies used high-intensity interval training (HIIT), two studies used progressive exercises, and two studies did not specify the type of exercise. The exercise intervention occurred 2 or 3 times each week. The control group intervention included conventional nursing, rehabilitation, health education, gentle stretching, cognitive therapy, and attention management. The characteristics of the interventions in the included studies are displayed in Table 2.

**Table 2:** Intervention characteristics of the included studies.

Authors	Intervention type	Frequency	Duration	Intensity	Control	Time points assessed	Outcomes
Deijle et al. (20)	Aerobic and strength training	Aerobic:2/week, strength: 3/week	12 weeks	1 h	Standard care	Baseline, 12 months, 24 months	MOCA\HADS
El-Tamawy et al. (21)	Aerobic exercise	3/week	8 weeks	40–45 min	Physiotherapy program	Baseline, 8 weeks	ACER
Gjellesvik et al. (22)	HIIT	3/week	8 weeks	NR	Standard care	3 months, 6 months, 10 months	6MWT\BBS\HADS\MoCA\SIS\
Harrington et al. (23)	Mixed exercise intervention	2/week	8 weeks	1 h	Standard care	Baseline, 9 weeks, 16 weeks	WHOQoL-Bref\HADS
Holmgren et al. (24)	Physical exercise	7/week	5 weeks	1 h	Educational discussion	Baseline, 5 weeks, 3 months, 6 months	GDS-15\SF-36\HRQoL
Ihle-Hansen et al. (25)	Physical exercise	2-3/week	18 months	30 min	Usual care	Baseline, 18 months	HADS\MMSE
Jiang et al. (26)	Aerobic exercise	NR	6 months	25 min	Health education and rehabilitation training	Baseline, 6 months	MOCA\SS-QOL
Koch et al. (27)	Aerobic and resistance training	3/week	12 weeks	100 min	Mild stretching and range-of-motion exercises	Baseline, 3 months	MOCA\CES-D\SIS\6MWT
Krawcyk et al. (28)	HIIT	5/week	12 weeks	3 × 3 min	Usual care	Baseline, 3 months	MoCA
Lai et al. (3)	Progressive exercise	3/week	3 months	NR	Usual care	Baseline, 3 months, 9 months,	GDS-15\BBS\SIS\SF-36
Lapointe et al. (29)	Aerobic exercise and HIIT	3/week	6 months	30 min	Usual care	Baseline, 6 months, 12 months	MOCA
Maeneja et al. (19)	Aerobic physical exercise	3/week	12 weeks	60 min	Walking and cognitive tasks	Baseline, 12 weeks	MMSE
Mead et al. (30)	Mixed exercise intervention	3/week	12 weeks	75 min	Seated relaxation	Baseline, 3 months, 7 months,	SF-36\HADS
Moore et al. (31)	Mixed exercise intervention	3/week	19 weeks	45–60 min	Home stretching program	Baseline, 20 weeks	6MWT\BBS\ACE-R\SIS
Sims et al. (32)	Progressive exercise	2/week	10 weeks	NR	Usual care	Baseline, 10 weeks, 6 months	CES-D\SF-12\AQOL
Song et al. (33)	Tai Chi	2/week	6 months	50 min	Symptom management program	Baseline, 3 months, 6 months	K-MOCA\K-MMSE\BBS\ADL\SS-QOL
Vahlberg et al. (4)	Mixed exercise intervention	2/week	3 months	75 min	Regular activities	Baseline, 3 months, 6 months, 15 months	BBS\6MWT\EQ-5D\GDS-20
Zedlitz et al. (34)	Mixed exercise intervention	2/week	12 weeks	2 h	Cognitive therapy	Baseline, 12 weeks, 6 months	HADS\6MWT\SA-SIP
Zhao et al. (35)	Tai Chi	NR	12 weeks	30 min	Attention control group	Baseline, 1 weeks, 8 weeks, 12 weeks, 16 weeks	BBS\ADL\GDS-SF\NIHSS\QOL\SSQOL
Zheng et al. (36)	Baduanjin	3/week	24 weeks	40 min	Routine medical or rehabilitative treatment	Baseline, 8 weeks, 16 weeks, 24 weeks, 28 weeks	MoCA\ADL

### 3.3 Risk-of-bias assessment

**Figure 2:**
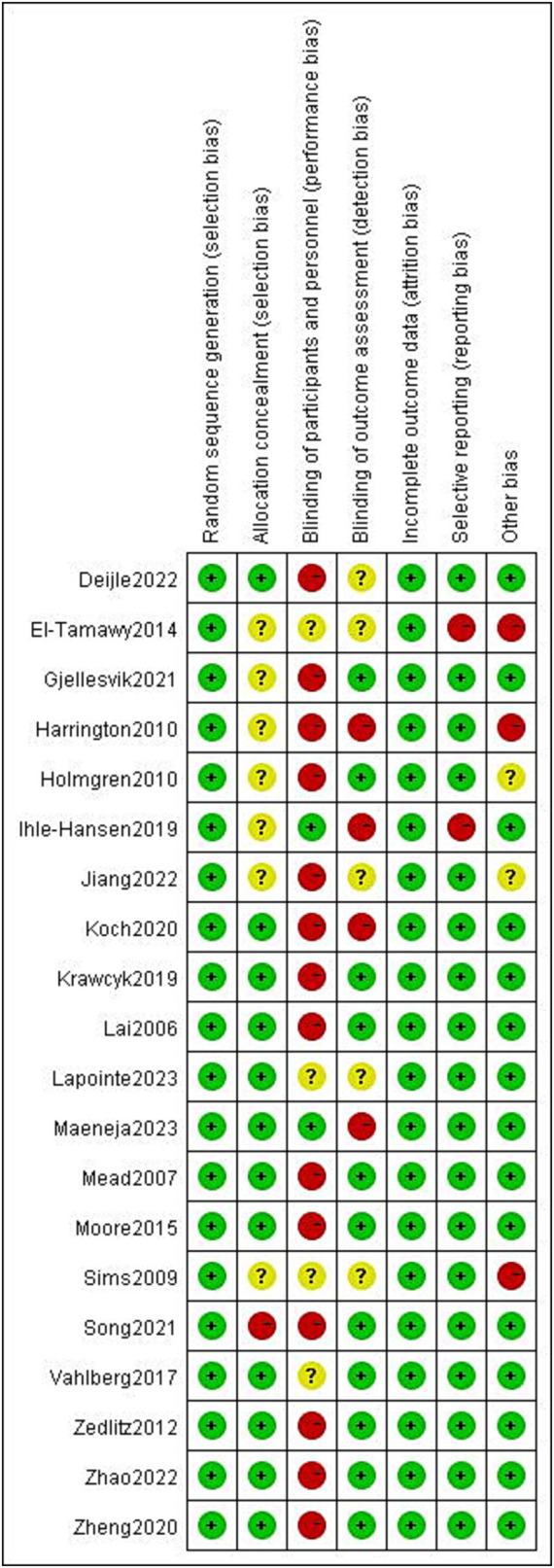
Risk of bias summary.

**Figure 3:**
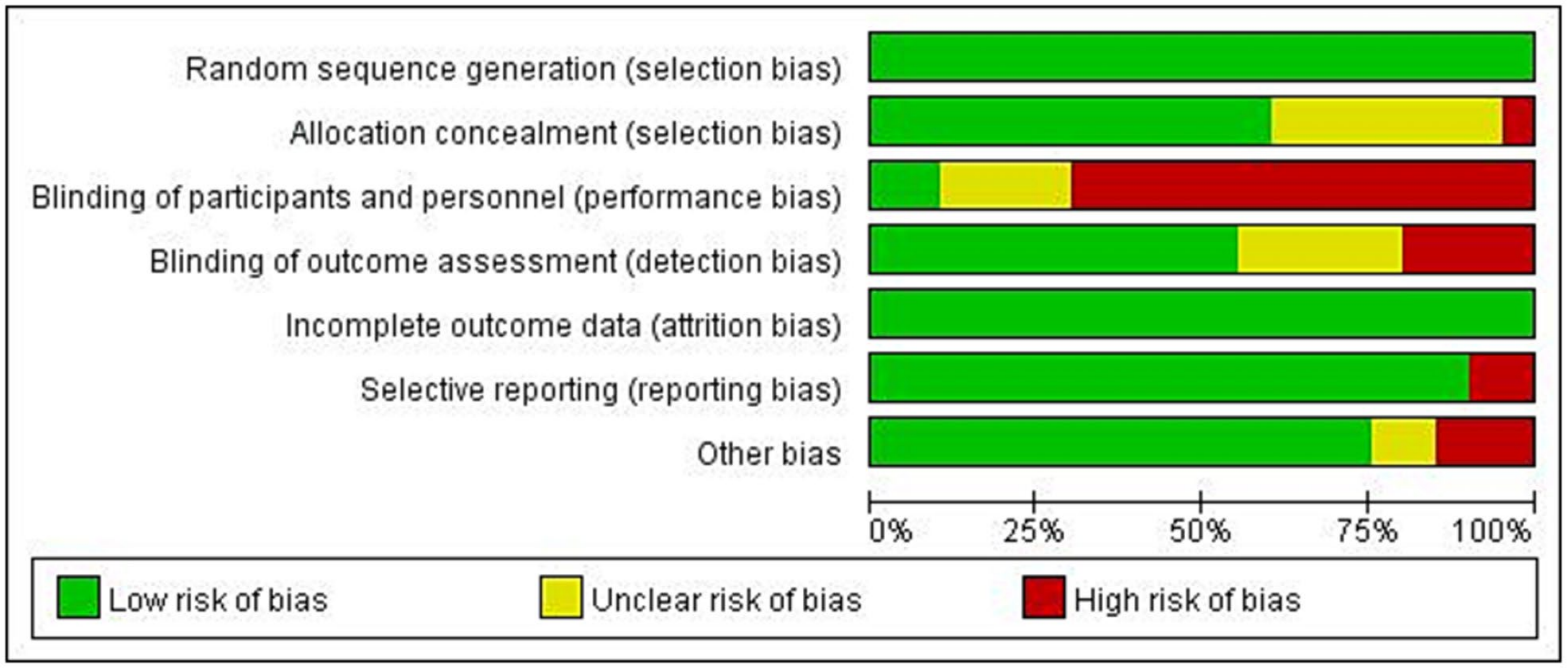
Risk of bias graph.

The results of the risk of bias assessment are summarized as depicted in Figure 2. The percentages of studies with low, unclear and high risk of bias were as follows: random sequence generation (100, 0, and 0%, respectively); allocation concealment (60, 35, and 5%, respectively); blinding of participants and personnel (10, 20, and 70%, respectively); blinding of outcome assessors (55, 25, and 20, respectively); incomplete outcome (100, 0, and 0%, respectively), selective outcome reporting (90, 0, and 10, respectively), and other bias (80, 5, and 15%, respectively). Detailed information regarding the risk of bias for the included studies is shown in Figure 3.

### 3.4 Meta-analysis findings: effects of exercise intervention

Among the 20 studies, we were interested in outcomes including depressive symptoms, cognitive function, physical function, and quality of life. The analysis considered various exercise times and detection scales of these two aspects. The exercise time was divided into two stages: longer than 3 weeks and less than or equal to 3 weeks. The Geriatric Depression Scale (GDS), the Hospital Anxiety and Depression Scale (HADS), the Hamilton Scale, and the Center for Epidemiologic Studies Depression Scale (CES-D) were among the instruments used to assess depressive symptoms. Cognition was measured using the Addenbrooke’s Cognitive Examination—Revised (ACE-R), the Mini-Mental State Examination (MMSE), and the Montreal Cognitive Assessment (MoCA). The physical function was evaluated using the 6-Minute Walk Test (6MWT) and the Berg Balance Scale (BBS). The following is our analysis of each result.

#### 3.4.1 Depression symptoms after stroke

**Figure 4:**
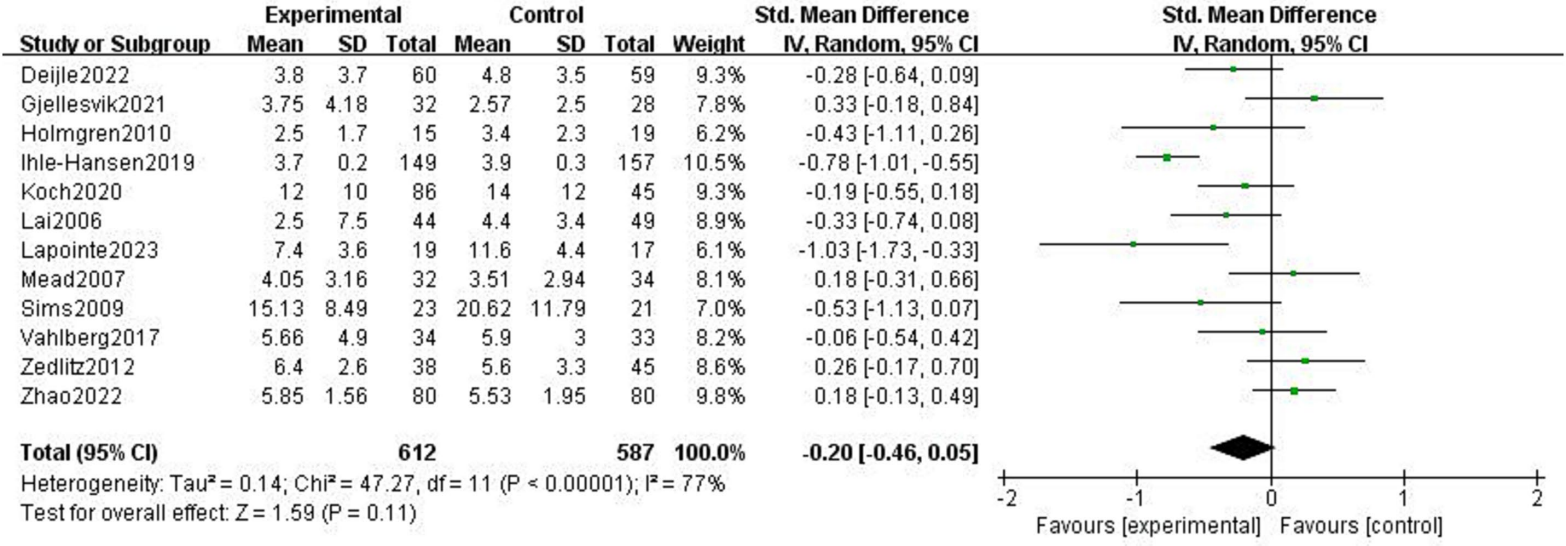
Effect of the exercise intervention on depressive symptoms.

Twelve research studies examined how exercise therapies affected stroke patients’ depressed symptoms (3, 4, 20–29). Due to study heterogeneity (*p* < 0.01, *I*^2^ = 77%), SMD and a random effects model were employed. Following the exercise intervention, the meta-analysis showed that there were no significant differences in depressive symptoms (*p* = 0.11, SMD = −0.2, 95% CI = −0.46–0.05; Figure 4).

**Figure 5:**
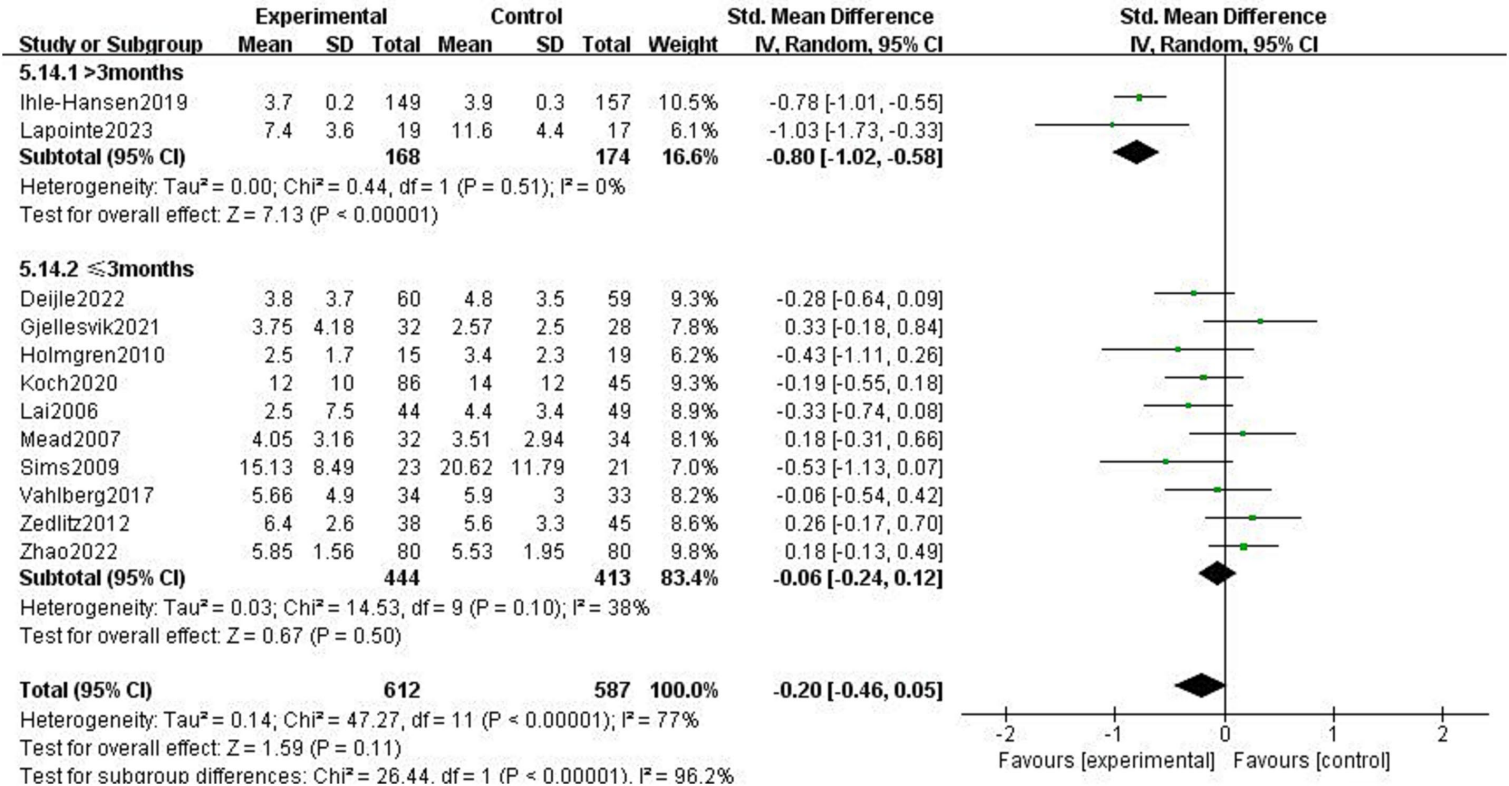
Effect of different motor durations on depressive symptoms.

**Figure 6:**
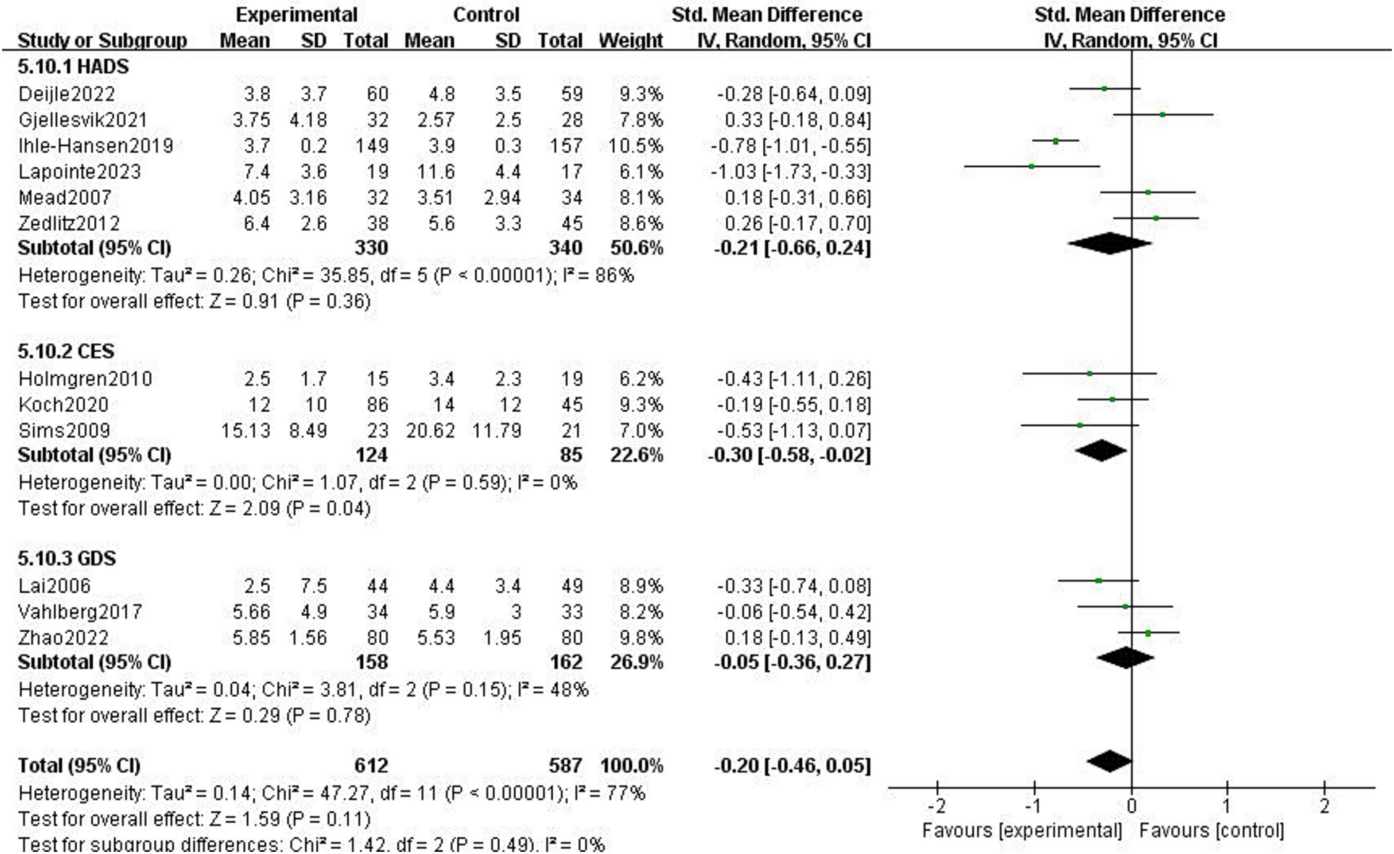
Effect of different detection tools on depressive symptoms.

The impact of exercise intervention on depression symptoms in stroke patients was highly heterogeneous. There may be hidden variables impacting this result. Therefore, we performed subgroup analysis using various scales and exercise intervention times. As shown in Figure 5, subgroup analysis based on exercise time showed significant differences among subgroups (*p* < 0.01). Exercise intervention time above 3 months was significantly different for depressive symptoms (SMD = −0.8, 95% CI = −1.02–0.58, *p* < 0.01, *I*^2^ = 0%), instead, when exercise intervention time was 3 months, there was no significant effect (SMD = −0.06, 95% CI = −0.24–0.12, *p* = 0.50, *I*^2^ = 38%). Subgroup analysis was performed according to the detection scale, with no significant difference between the subgroups (*p* = 0.49, *I*^2^ = 0%; Figure 6).

#### 3.4.2 Cognitive function after stroke

**Figure 7:**
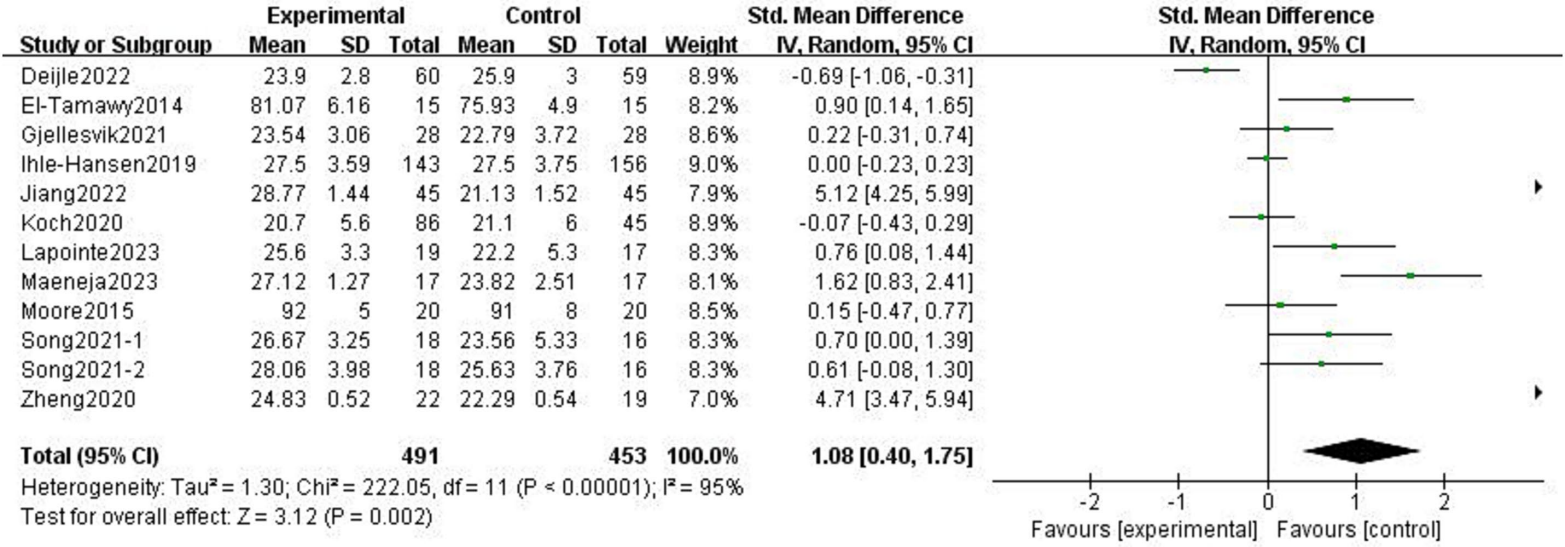
Effect of the exercise intervention on cognitive function.

The effects of exercise interventions on the cognitive function of stroke patients were documented in 11 research studies (19–21, 23–25, 30–34). Similar to the study by Song et al. (33) in which the authors tested two scales, we divided it into two parts for the meta-analysis. SMD was selected as the effect size combination in a random effects model due to study heterogeneity (*p* < 0.01, *I*^2^ = 95%). The meta-analysis showed that cognitive performance improved after exercise intervention (*p* = 0.002, SMD = 1.08, 95% CI = 0.40–1.75; Figure 7).

**Figure 8:**
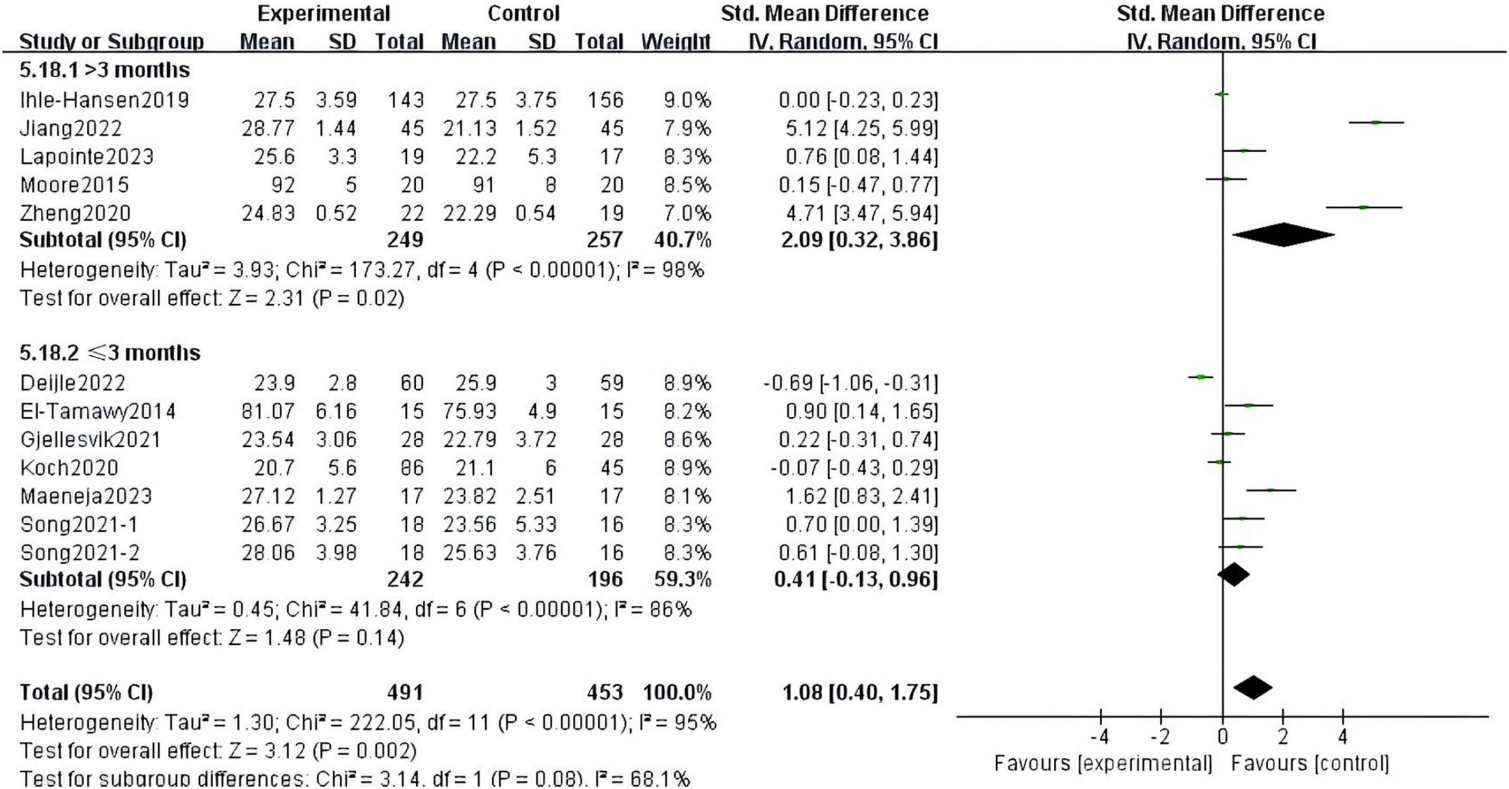
Effect of different motor durations on cognitive function.

**Figure 9:**
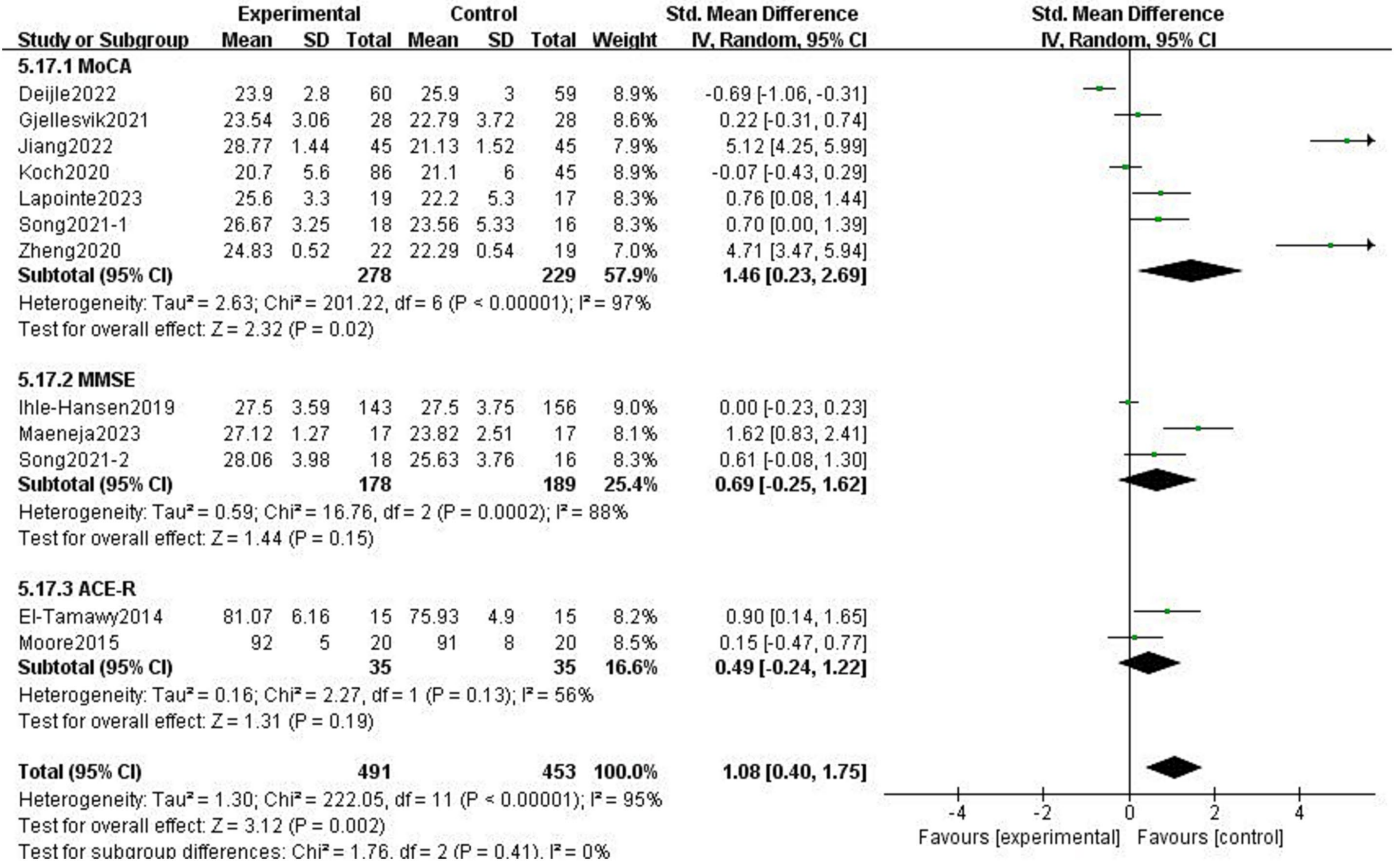
Effect of different detection tools on cognitive function.

There was heterogeneity among the groups, indicating that underlying factors might have an impact on how exercise interventions affect stroke patients’ cognitive function. Because the included studies involved different exercise intervention times and a variety of different instrumental tests, we performed a subgroup analysis of these two variables. Subgroup analysis based on exercise duration, with no significant differences between the subgroups (*p* = 0.08, *I*^2^ = 68.1%; Figure 8). Subgroup analysis was carried out using the detection scale, with no significant differences between the subgroups (*p* = 0.41, *I*^2^ = 0%; Figure 9).

#### 3.4.3 Physical function after stroke

##### 3.4.3.1 BBS

**Figure 10:**
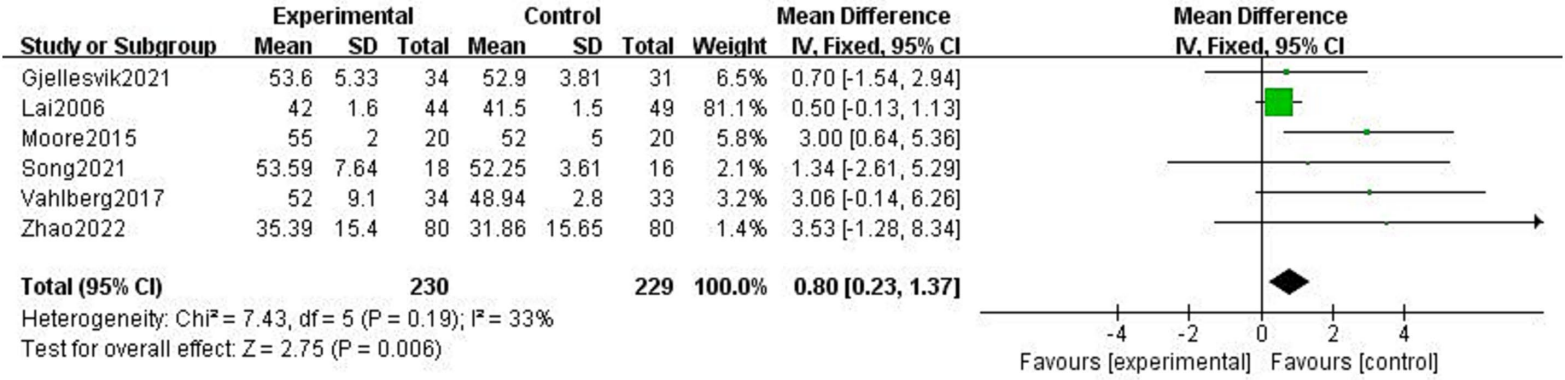
Effect of the exercise intervention on BBS.

The BBS was used to balance body dynamics with static balance, and it included 14 items with a total score of 56. Six studies reported the impact of exercise intervention on stroke patients’ balance, and the mean difference technique was used to assess each study (3, 4, 21, 29, 32, 33). The fixed-effect model was selected because of the low heterogeneity among the six studies (*p* = 0.19, *I*^2^ = 33%). The results demonstrated that the experimental group’s equilibrium function was greater than the control group’s and that the difference was statistically significant (MD = 0.80, 95% CI = 0.23 ~ 1.37, *p* < 0.01, Figure 10).

##### 3.4.3.2 6MWT

**Figure 11:**
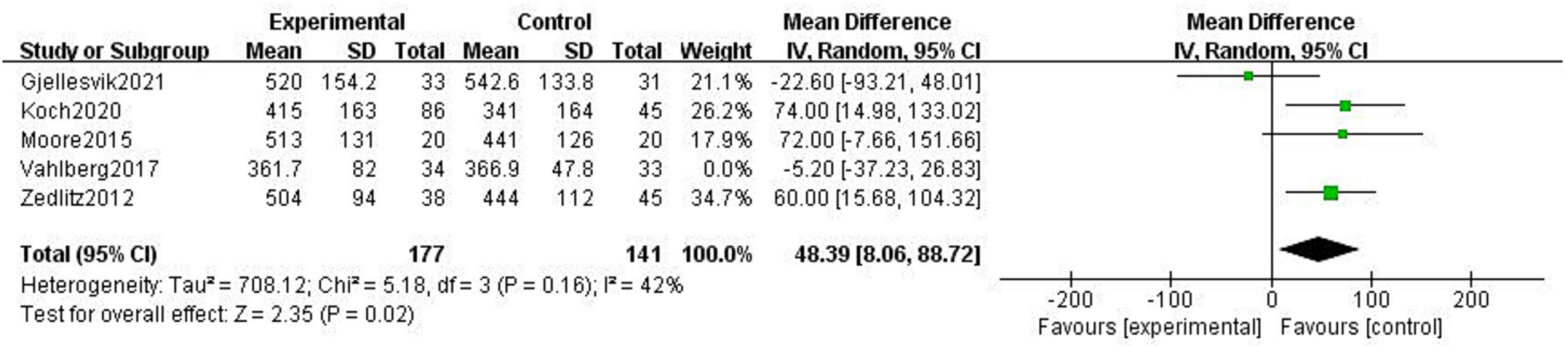
Effect of the exercise intervention on 6MWT.

The 6MWT is a valid tool for assessing physical endurance to record the total walking distance in meters over 6 min. Five studies examined how exercise interventions affected stroke patients’ 6MWT scores (4, 21, 24, 28, 32). With heterogeneity between studies (*p* = 0.11, *I*^2^ = 65%), a random-effects model was selected. By excluding the literature, one by one, we found that Vahlberg et al. (4) had a great impact on heterogeneity. After removing Vahlberg et al. (4), heterogeneity decreased (*p* = 0.02, *I*^2^ = 42%). There was a statistically significant difference between the experimental and control groups’ walking distances (MD = 48.39, 95% CI = 8.06–88.72, *p* = 0.02, Figure 11).

#### 3.4.4 Quality of life after stroke

A total of 12 studies included at least one measure of quality of life, and 3 studies included 2 measures (27, 29, 33). Due to substantial differences in outcome evaluation indicators across various scales that assess quality of life, a meta-analysis could not be conducted; consequently, the results were described statistically. One study used EuroQol five dimensions questionnaire (EQ-5D), revealing any significance between-group changes at follow-up (4). Five studies reported the results of the evaluation of the Stroke Impact Scale (SIS), and three of them reported that exercise improves patients’ quality of life, including their ability to regain their emotional and cognitive capacities (3, 27, 32). At the same time, 2 reported that the groups did not differ significantly on the SIS scale for the remaining outcome variables in the exercise and non-exercise groups (21, 24). Three studies reported the results of the evaluation of the Stroke-Specific Quality of Life scale (SS-QOL) (29, 31, 33), and 3 studies reported the results of the evaluation of the Activity of Daily Living scale (ADL) (29, 33, 34), these studies collectively indicated that exercise could enhance the quality of life of patients. Three studies reported the evaluation results of the Short Form Survey Scale (SF) (22, 26, 27). One article showed the beneficial effects of exercise (26), and two studies did not show a favorable effect on the evaluation of the SF scale (22, 27). One article reported the Assessment of Quality of Life (A-QOL), and one article reported the World Health Organization Quality of Life (WHOQOL). According to a study, baseline group differences in AQoL, social support, recovery locus of control, and life satisfaction scores were non-existent (27). At 6 months, there was evidence that the intervention group had improved more in the psychological area of the WHOQOL-BREF (35).

### 3.5 Safety

Among the 20 studies, 8 studies did not describe adverse events, 12 studies described adverse events, and 9 of them had no adverse events. Adverse events occurred in three studies, and one study (36) reported one adverse event, but it was not related to the intervention. One study (26) reported fall events, and 11 of them were reported in the exercise group, but all occurred outside the exercise intervention time. One study (24) reported that the most common adverse events of exercise interventions were musculoskeletal disorders, infections, and blood pressure abnormalities.

### 3.6 Sensitivity analysis and publication bias

**Figure 12:**
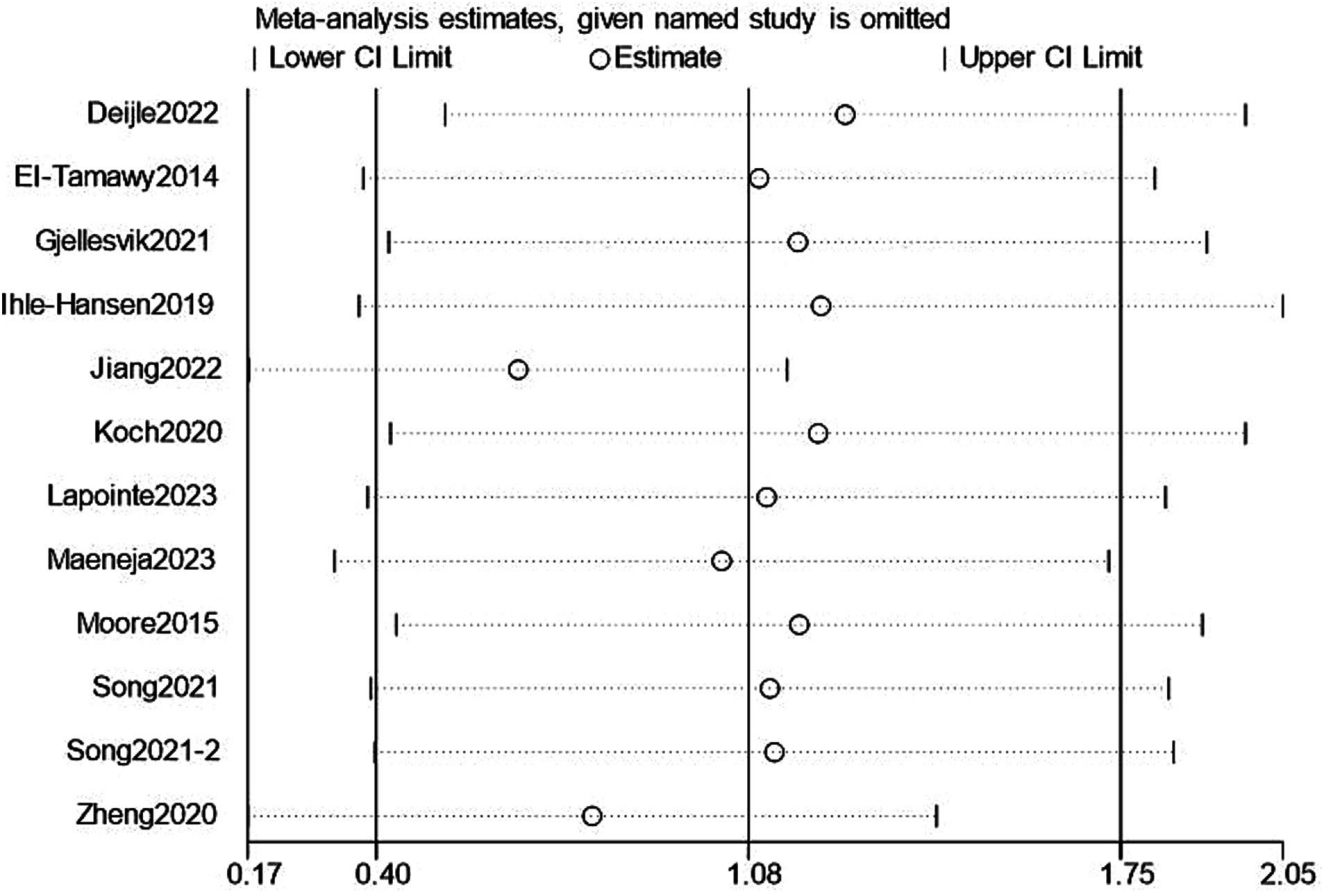
Sensitivity analysis for cognition.

**Figure 13:**
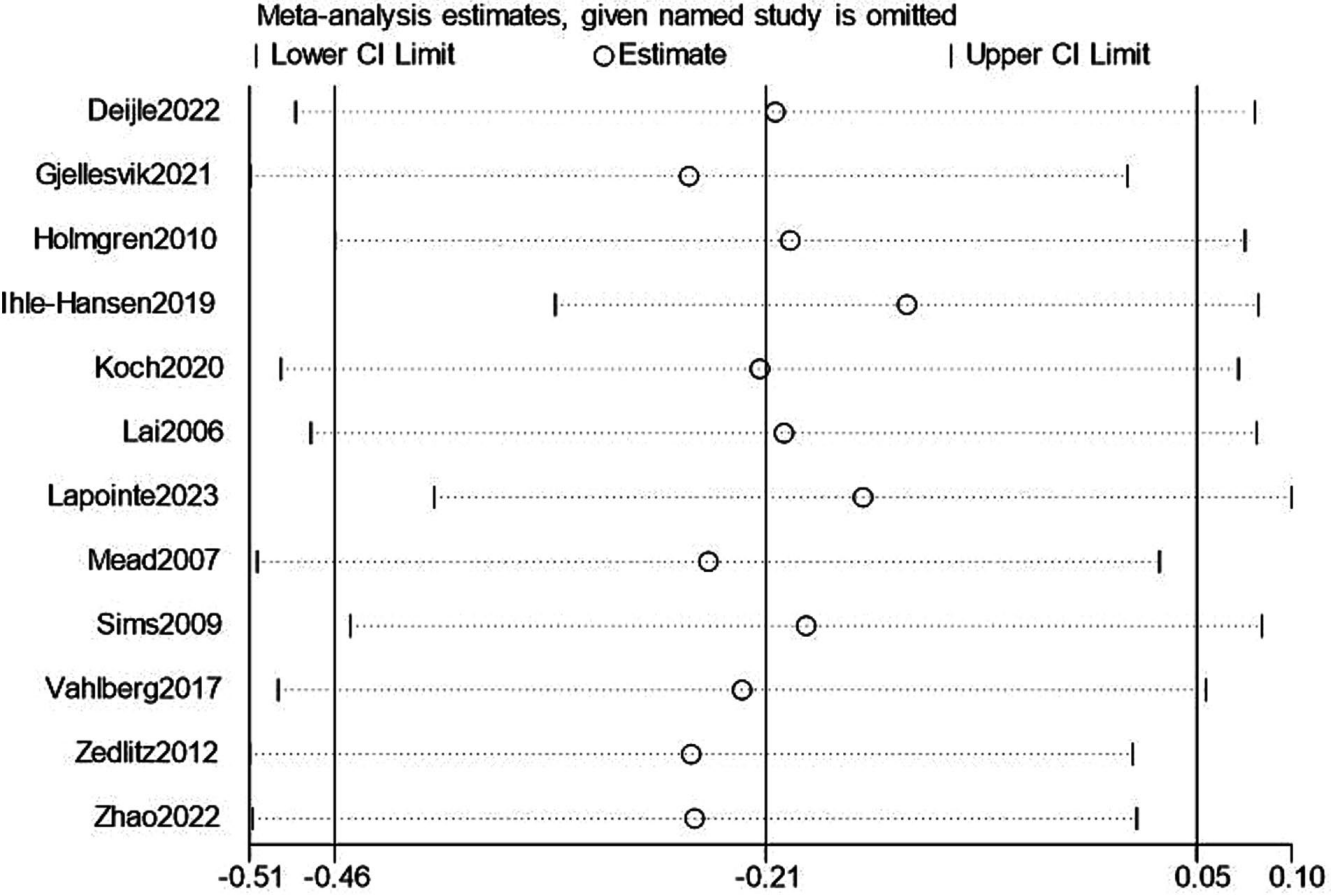
Sensitivity analysis for depression.

We performed a leave-one-out sensitivity analysis examining cognitive and depression scores; excluding individual studies did not change the results. Additionally, there was no discernible difference in the combined estimates’ direction or magnitude, indicating that our study was stable and reliable, as shown in Figures 12, 13.

### 3.7 GRADE certainty of evidence

**Figure 14:**
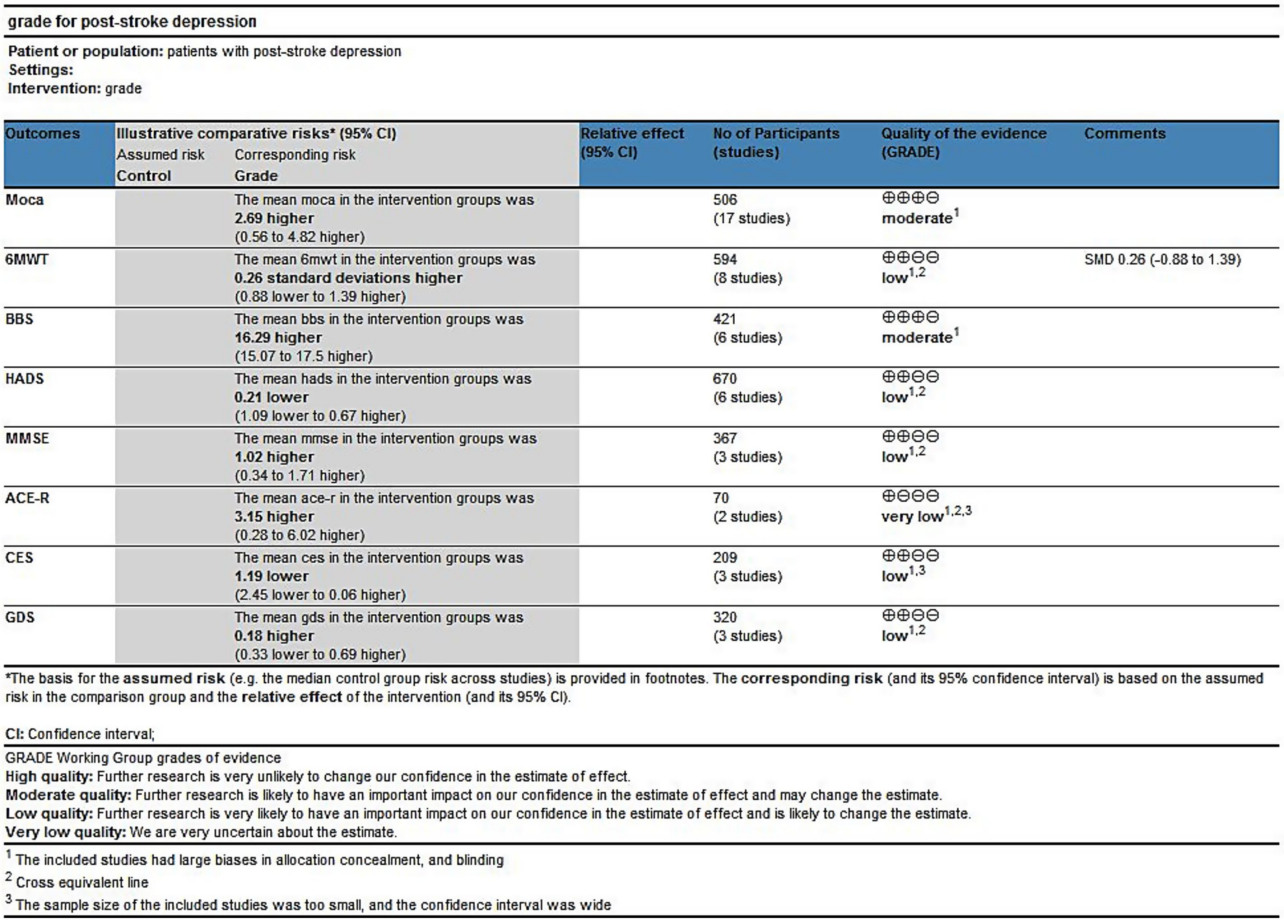
GRADE for the quality of evidence of outcome indicators.

Although all included studies were randomized controlled trials, allocation concealment, and blinding could not be achieved due to the nature of the intervention method, which was an exercise intervention. As a result, the strength of evidence was downgraded by one level; only the strength of MoCA and BBS evidence was considered moderate. According to the results of the forest plot, some research indicators crossed the equivalent line, indicating that there was no significant difference in the effect of exercise intervention, and the strength of evidence was downgraded by one level. Therefore, the strength of evidence of 6MWT, HADS, MMSE, and GDS was considered low. The assessments were subject to inaccuracies. The strength of evidence for ACE-R was considered very low because of the small sample size and the forest plot results crossing the equivalence line. Complete GRADE assessments for all treatments are shown in Figure 14.

## 4 Discussion

This systematic review aimed to evaluate how exercise interventions affect cognitive function, depressive symptoms, physical function, and quality of life after a stroke. The results indicated that exercise interventions could enhance the cognitive and physical functions of stroke patients. However, the results also showed that short-term exercise interventions had no significant impact on depressive symptoms. The exercise duration should be more than 3 months to effectively alleviate and improve depressive symptoms.

Exercise as a complementary therapy, to improve the stroke sequelae there is some value. There is evidence that organized sports for short-term and long-term function after stroke (37). Exercise can alter metabolism and regulate cerebrovascular control in the short term, thereby reducing stroke recurrence and improving cardiovascular and cerebrovascular health, with long-term benefits. The American Stroke Association, in a scientific statement, suggested that sports should be included in the management of stroke survivors (38). Repeated, long-term exercise can promote the production of neurons, glia, synapses, and blood vessels, and these changes favor the improvement of stroke sequelae (39–41). Exercise interventions are complex and usually involve different durations, types, frequencies, and intensity of exercises. In patients with stroke, an appropriate exercise prescription is the foundation and guarantee of sports training. It is unclear how the treatment effect of varying exercise duration and the sensitivity of various detection scales to the results differ, even though numerous studies have examined cognitive performance and depressive symptoms in stroke patients following exercise. A comprehensive meta-analysis was performed in this study, which divided exercise duration into >3 months and ≤ 3 months. Since different scales evaluated cognitive function and depressive symptoms, subgroup analysis was performed by evaluation scale.

Exercise intervention does not significantly improve the depressive symptoms of stroke patients. The meta-analysis’s results demonstrated that there was no discernible difference in depression symptoms between detection scales. However, the subgroup meta-analysis showed that the intervention with a longer duration of >3 months was more beneficial on depressive symptoms than the intervention with a duration of ≤3 months. Therefore, we found that exercise duration may be the influencing factor of whether depressive symptoms can be improved after exercise intervention in stroke patients. The positive impact of the right exercise duration in alleviating depressive symptoms may be associated with physiological mechanisms. This is in agreement with the results of previous studies, which demonstrated that exercise can reshape the brain structure of patients with depression (42), activate the function of pertinent brain regions (43), and motivate behavioral adaptation changes (44), thereby improving the brain neural processing of patients with depression and delaying cognitive degradation. However, the shorter exercise duration may not be sufficient to trigger these physiological responses to establish a regular physiological rhythm that can stimulate the brain to produce more neural connections and remodeling, making the effect of alleviating depression insufficiently sustained and obvious (45).

Our results demonstrate that stroke patients’ cognitive function can be enhanced by exercise intervention. However, there were differences between studies, and subgroup analyses were performed on exercise duration and detection scales. Exercise duration and detection scales did not account for the high heterogeneity in cognitive function. The high heterogeneity may have been due to other factors, including different exercise interventions and the wide variation in the timing of stroke among patients.

Improving balance is an essential goal in stroke treatment, a strong predictor of functional recovery (46) and walking capacity (47), as well as an important factor in reducing the occurrence of falls after stroke. To effectively lower the incidence of limb hemiplegia and atrophy, patients can benefit from repeated strengthening exercises that enhance muscle tension and body coordination and aid in limb rehabilitation (48). Exercise can help stroke patients get more balanced, as evidenced by the fact that the exercise group’s BBS scores were higher than that of the control group. As for walking ability, the results of 6MWT after removing Vahlberg et al. (4) revealed that the 6MWT score of the exercise group was more significant than the control group, indicating that exercise intervention can improve the walking ability of stroke patients. By comparing the research variables in the literature, we found that the population included in Vahlberg et al. (4) was older adults while there was no difference in other exercise types, frequency, cycle, and the number of participants compared with other literatures.

This research includes literature from 12 studies involving the influence of exercise intervention on the quality of life of patients with cerebral apoplexy. Nine of these studies demonstrate that exercise intervention can improve the quality of life of patients with stroke. In general, the cognitive function and physical function and increasing the quality of life of patients with cerebral apoplexy were positively correlated, improve cognitive function and body function can improve the patient’s awareness and ability to adapt to the outside world, thus improving the quality of life of patients with cerebral apoplexy (49).

This study noted some heterogeneity since data from several studies were gathered for analysis. This heterogeneity can be explained by several factors, including the fact that the included studies were conducted between 2006 and 2023, that the patients came from a variety of nations, including the United States, China, Australia, and others, and that their sociocultural context may have had an impact. Furthermore, studies included patients who had strokes at various times in their lives, which would have added to the results’ heterogeneity.

## 5 Future implications

This review of the results demonstrated that exercise intervention is beneficial for rehabilitating both cognitive and physical function in stroke patients. At the same time, more than 3 months of continuous exercise could help stroke patients improve depressive symptoms. Therefore, encouraging patients to engage in long-term, persistent physical activity can help prevent and reduce stroke sequelae. It is worth noting that stroke patients should be accompanied and supervised by professionals during exercise to prevent adverse events, while ensuring patient compliance and the effectiveness of the exercise. Within the studies encompassed in this review, the mean exercise duration was approximately 1 h, and the most common exercise frequency was 3 times per week. Notably, these findings are in accordance with the guidelines and recommendations established by the UK National Institute for Health and Care Excellence (NICE) for both clinical and non-clinical groups (50). The duration of exercise was not less than 3 months. However, given the substantial variability in exercise intensity among these investigations, it is difficult to draw definitive conclusions about the optimal exercise intensity in the current review.

## 6 Limitations

This system research has certain limitations: first, this study on cerebral apoplexy patients to limit time and type of stroke may affect the study results. Second, while the subgroup analysis of athletic time, the motion frequency, and dose could also lead to larger heterogeneity, which should not be ignored. Third, due to the nature of the exercise intervention, participants in the blind method are very difficult, so the subjective rating may be affected by the placebo effect. Fourth, only English literature may not be able to cover the whole range of existing research. Given these limitations, the results of this comprehensive review should be carefully explained.

## 7 Conclusion

The results showed that the cognitive function, balance, walking speed, and quality of life of stroke patients were improved after exercise intervention, and more prolonged exercise duration (>3 months) helped to improve the depressive symptoms of stroke patients. Consequently, we advocate that stroke patients partake in physical exercise 3 times a week for 1 h each time. The exercise should continue for no less than 3 months to ensure the best therapeutic effect. Moreover, the determination of exercise intensity should be a personalized process, carefully tailored to align with each patient’s unique physical capabilities and limitations.

## Data Availability

The original contributions presented in the study are included in the article/Supplementary material, further inquiries can be directed to the corresponding authors.

## Glossary

ACE-R: Addenbrooke’s Cognitive Examination—Revised
ADL: Activity of Daily Living scale
A-QOL: Assessment of Quality of Life
BBS: Berg Balance Scale
CES-D: Center for Epidemiologic Studies Depression Scale
CNKI: Chinese National Knowledge Infrastructure
EQ-5D: EuroQol five dimensions questionnaire
GDS: Geriatric Depression Scale
GRADE: Grading Assessment Development, and Evaluation
HADS: Hospital Anxiety and Depression Scale
HIIT: high-intensity interval training
HRQoL: Health-related quality of life
K-MMSE: Korean-Mini-Mental State Examination
K-MOCA: Korean-Montreal Cognitive Assessment
MD: Mean difference
MMSE: Mini-Mental State Examination
MoCA: Montreal Cognitive Assessment
6MWT: 6-Minute Walk Test
NICE: National Institute for Health and Care Excellence
NIHSS: NIH Stroke Scale
PRISMA: Preferred Reporting Items for Systematic Reviews and Meta-Analyses
PROSPERO: Prospective Register of Systematic Reviews
PSD: post-stroke depression
QOL: Quality of Life scale
SA-SIP: The Stroke-Adapted Sickness Impact Profile
SF: Survey scale
SF-36: Physical Component Summary of the Short Form 36
SIS: Stroke Impact Scale
SMD: standard mean difference
SS-QOL: Stroke-Specific Quality of Life scale
VIPC: China Science and Technology Journal Database
WHOQOL: World Health Organization Quality of Life
WHOQOL-BREF: World Health Organization Quality of Life short version of Life
