# Supplementary material for: The effect of exercise interventions on reducing the risk of depressive and cognitive disorders in post-stroke—a systematic review and meta-analysis
Source: Front Neurol. 2025 Mar 24;16:1564347. doi: 10.3389/fneur.2025.1564347 (PMC11973079; doi:10.3389/fneur.2025.1564347)
Supplement: Supplementary file 1 [file Data_Sheet_1.docx]

**Supplemental Online Content**

Appendix 1. Search strategy in for different database (from inception to August 30, 2024).

| Database | Search strategy |
| --- | --- |
| PubMed | #1(((((("Cognition"[Mesh]) OR (Cognitions[Title/Abstract])) OR (Cognitive Function[Title/Abstract])) OR (Cognitive Functions[Title/Abstract])) OR (Function, Cognitive[Title/Abstract])) OR (Functions, Cognitive[Title/Abstract]) |
|  | #2((((("Depression"[Mesh]) OR (Depressive Symptoms[Title/Abstract])) OR (Depressive Symptom[Title/Abstract])) OR (Symptom, Depressive[Title/Abstract])) OR (Emotional Depression[Title/Abstract])) OR (Depression, Emotional[Title/Abstract]) |
|  | #3 (((((((("Anxiety"[Mesh]) OR (Angst[Title/Abstract])) OR (Social Anxiety[Title/Abstract])) OR (Anxieties, Social[Title/Abstract])) OR (Anxiety, Social[Title/Abstract])) OR (Social Anxieties[Title/Abstract])) OR (Hypervigilance[Title/Abstract])) OR (Nervousness[Title/Abstract])) OR (Anxiousness[Title/Abstract]) |
|  | #4 #2 OR #3 |
|  | #5((("Sports"[Mesh]) OR (Sport[Title/Abstract])) OR (Athletics[Title/Abstract])) OR (Athletic[Title/Abstract]) |
|  | #6 ((((((((((exercise[MeSH Terms]) OR (Circuit-Based Exercise[MeSH Terms])) OR (resistance training[MeSH Terms])) OR (muscle stretching exercises[MeSH Terms])) OR (sports[MeSH Terms])) OR (physical education and training[MeSH Terms])) OR (walking[MeSH Terms])) OR (motor activity[MeSH Terms])) OR (physical fitness[MeSH Terms])) OR (((((((((((((((exercis*[Title/Abstract]) OR (exerciz*[Title/Abstract])) OR (weight bearing[Title/Abstract])) OR (dancing[Title/Abstract])) OR (dance therapy[Title/Abstract])) OR (stair climb*[Title/Abstract])) OR (treadmill*[Title/Abstract])) OR (walk[Title/Abstract])) OR (walking[Title/Abstract])) OR (swim*[Title/Abstract])) OR (yoga[Title/Abstract])) OR (pilates[Title/Abstract])) OR (tai chi[Title/Abstract])) OR (postural balance[Title/Abstract])) OR (body equilibrium[Title/Abstract]))) |
|  | #7((((((((((((((((((((((((("Exercise"[Mesh])OR (Exercises[Title/Abstract])) OR (Physical Activity[Title/Abstract])) OR (Activities, Physical[Title/Abstract])) OR (Activity, Physical[Title/Abstract])) OR (Physical Activities[Title/Abstract])) OR (Exercise, Physical[Title/Abstract])) OR (Exercises, Physical[Title/Abstract])) OR (Physical Exercise[Title/Abstract])) OR (Physical Exercises[Title/Abstract])) OR (Acute Exercise[Title/Abstract])) OR (Acute Exercises[Title/Abstract])) OR (Exercise, Acute[Title/Abstract])) OR (Exercises, Acute[Title/Abstract])) OR (Exercise, Isometric[Title/Abstract])) OR (Exercises, Isometric[Title/Abstract])) OR (Isometric Exercises[Title/Abstract])) OR (Isometric Exercise[Title/Abstract])) OR (Exercise, Aerobic[Title/Abstract])) OR (Aerobic Exercise[Title/Abstract])) OR (Aerobic Exercises[Title/Abstract])) OR (Exercises, Aerobic[Title/Abstract])) OR (Exercise Training[Title/Abstract])) OR (Exercise Trainings[Title/Abstract])) OR (Training, Exercise[Title/Abstract])) OR (Trainings, Exercise[Title/Abstract]) |
|  | #8 #5 OR #6 OR #7 |
|  | #9(((((((((((((((((((((((((((("Stroke"[Mesh]) OR (Strokes[Title/Abstract])) OR (Cerebrovascular Accident[Title/Abstract])) OR (Cerebrovascular Accidents[Title/Abstract])) OR (CVA (Cerebrovascular Accident[Title/Abstract]))) OR (CVAs (Cerebrovascular Accident[Title/Abstract]))) OR (Cerebrovascular Apoplexy[Title/Abstract])) OR (Apoplexy, Cerebrovascular[Title/Abstract])) OR (Vascular Accident, Brain[Title/Abstract])) OR (Brain Vascular Accident[Title/Abstract])) OR (Brain Vascular Accidents[Title/Abstract])) OR (Vascular Accidents, Brain[Title/Abstract])) OR (Cerebrovascular Stroke[Title/Abstract])) OR (Cerebrovascular Strokes[Title/Abstract])) OR (Stroke, Cerebrovascular[Title/Abstract])) OR (Strokes, Cerebrovascular[Title/Abstract])) OR (Apoplexy[Title/Abstract])) OR (Cerebral Stroke[Title/Abstract])) OR (Cerebral Strokes[Title/Abstract])) OR (Stroke, Cerebral[Title/Abstract])) OR (Strokes, Cerebral[Title/Abstract])) OR (Stroke, Acute[Title/Abstract])) OR (Acute Stroke[Title/Abstract])) OR (Acute Strokes[Title/Abstract])) OR (Strokes, Acute[Title/Abstract])) OR (Cerebrovascular Accident, Acute[Title/Abstract])) OR (Acute Cerebrovascular Accident[Title/Abstract])) OR (Acute Cerebrovascular Accidents[Title/Abstract])) OR (Cerebrovascular Accidents, Acute[Title/Abstract]) |
|  | #10 (((((((((((((("Basal Ganglia Cerebrovascular Disease"[Mesh]) OR (Vascular Diseases, Basal Ganglia[Title/Abstract])) OR (Vascular Disease, Basal Ganglia[Title/Abstract])) OR (Basal Ganglia Vascular Disease[Title/Abstract])) OR (Cerebrovascular Disease, Basal Ganglia[Title/Abstract])) OR (Lenticulostriate Vasculopathy[Title/Abstract])) OR (Lenticulostriate Vasculopathies[Title/Abstract])) OR (Vasculopathies, Lenticulostriate[Title/Abstract])) OR (Vasculopathy, Lenticulostriate[Title/Abstract])) OR (Lenticulostriate Vascular Diseases[Title/Abstract])) OR (Lenticulostriate Vascular Disease[Title/Abstract])) OR (Vascular Disease, Lenticulostriate[Title/Abstract])) OR (Vascular Diseases, Lenticulostriate[Title/Abstract])) OR (Lenticulostriate Diseases, Vascular[Title/Abstract])) OR (Vascular Lenticulostriate Diseases[Title/Abstract]) |
|  | #11 ((((((((((((((((((((((((((((((("Brain Infarction"[Mesh]) OR (Brain Infarctions[Title/Abstract])) OR (Infarction, Brain[Title/Abstract])) OR (Infarctions, Brain[Title/Abstract])) OR (Brain Infarct[Title/Abstract])) OR (Brain Infarcts[Title/Abstract])) OR (Infarct, Brain[Title/Abstract])) OR (Infarcts, Brain[Title/Abstract])) OR (Anterior Circulation Brain Infarction[Title/Abstract])) OR (Infarction, Brain, Anterior Circulation[Title/Abstract])) OR (Infarction, Anterior Circulation, Brain[Title/Abstract])) OR (Anterior Circulation Infarction, Brain[Title/Abstract])) OR (Brain Infarction, Anterior Circulation[Title/Abstract])) OR (Venous Infarction, Brain[Title/Abstract])) OR (Brain Venous Infarction[Title/Abstract])) OR (Brain Venous Infarctions[Title/Abstract])) OR (Infarction, Brain Venous[Title/Abstract])) OR (Infarctions, Brain Venous[Title/Abstract])) OR (Venous Infarctions, Brain[Title/Abstract])) OR (Brain Infarction, Venous[Title/Abstract])) OR (Brain Infarctions, Venous[Title/Abstract])) OR (Infarction, Venous Brain[Title/Abstract])) OR (Infarctions, Venous Brain[Title/Abstract])) OR (Venous Brain Infarction[Title/Abstract])) OR (Venous Brain Infarctions[Title/Abstract])) OR (Anterior Cerebral Circulation Infarction[Title/Abstract])) OR (Infarction, Anterior Cerebral Circulation[Title/Abstract])) OR (Brain Infarction, Posterior Circulation[Title/Abstract])) OR (Posterior Circulation Infarction, Brain[Title/Abstract])) OR (Posterior Circulation Brain Infarction[Title/Abstract])) OR (Infarction, Brain, Posterior Circulation[Title/Abstract])) OR (Infarction, Posterior Circulation, Brain[Title/Abstract]) |
|  | #12((((((((((((((((((((((((((((((((("Paresis"[Mesh]) OR (Pareses[Title/Abstract])) OR (Muscular Paresis[Title/Abstract])) OR (Muscular Pareses[Title/Abstract])) OR (Pareses, Muscular[Title/Abstract])) OR (Paresis, Muscular[Title/Abstract])) OR (Muscle Paresis[Title/Abstract])) OR (Muscle Pareses[Title/Abstract])) OR (Pareses, Muscle[Title/Abstract])) OR (Paresis, Muscle[Title/Abstract])) OR (Monoparesis[Title/Abstract])) OR (Monopareses[Title/Abstract])) OR (Lower Extremity Paresis[Title/Abstract])) OR (Extremity Pareses, Lower[Title/Abstract])) OR (Extremity Paresis, Lower[Title/Abstract])) OR (Lower Extremity Pareses[Title/Abstract])) OR (Pareses, Lower Extremity[Title/Abstract])) OR (Paresis, Lower Extremity[Title/Abstract])) OR (Crural Paresis[Title/Abstract])) OR (Crural Pareses[Title/Abstract])) OR (Pareses, Crural[Title/Abstract])) OR (Paresis, Crural[Title/Abstract])) OR (Upper Extremity Paresis[Title/Abstract])) OR (Extremity Pareses, Upper[Title/Abstract])) OR (Extremity Paresis, Upper[Title/Abstract])) OR (Pareses, Upper Extremity[Title/Abstract])) OR (Paresis, Upper Extremity[Title/Abstract])) OR (Upper Extremity Pareses[Title/Abstract])) OR (Brachial Paresis[Title/Abstract])) OR (Brachial Pareses[Title/Abstract])) OR (Pareses, Brachial[Title/Abstract])) OR (Paresis, Brachial[Title/Abstract])) OR (Hemiparesis[Title/Abstract])) OR (Hemipareses[Title/Abstract]) |
|  | #13 (((((((((((((((((((((((((((("Hemiplegia"[Mesh]) OR (Hemiplegias[Title/Abstract])) OR (Hemiplegia, Transient[Title/Abstract])) OR (Hemiplegias, Transient[Title/Abstract])) OR (Transient Hemiplegia[Title/Abstract])) OR (Transient Hemiplegias[Title/Abstract])) OR (Monoplegia[Title/Abstract])) OR (Monoplegias[Title/Abstract])) OR (Hemiplegia, Post-Ictal[Title/Abstract])) OR (Hemiplegia, Post Ictal[Title/Abstract])) OR (Hemiplegias, Post-Ictal[Title/Abstract])) OR (Post-Ictal Hemiplegia[Title/Abstract])) OR (Post-Ictal Hemiplegias[Title/Abstract])) OR (Hemiplegia, Crossed[Title/Abstract])) OR (Crossed Hemiplegia[Title/Abstract])) OR (Crossed Hemiplegias[Title/Abstract])) OR (Hemiplegias, Crossed[Title/Abstract])) OR (Hemiplegia, Flaccid[Title/Abstract])) OR (Flaccid Hemiplegia[Title/Abstract])) OR (Flaccid Hemiplegias[Title/Abstract])) OR (Hemiplegias, Flaccid[Title/Abstract])) OR (Hemiplegia, Infantile[Title/Abstract])) OR (Hemiplegias, Infantile[Title/Abstract])) OR (Infantile Hemiplegia[Title/Abstract])) OR (Infantile Hemiplegias[Title/Abstract])) OR (Hemiplegia, Spastic[Title/Abstract])) OR (Hemiplegias, Spastic[Title/Abstract])) OR (Spastic Hemiplegia[Title/Abstract])) OR (Spastic Hemiplegias[Title/Abstract]) |
|  | #14((((((((((((((((((((((((("Cerebrovascular Disorders"[Mesh])OR (Cerebrovascular Disorder[Title/Abstract])) OR (Vascular Diseases, Intracranial[Title/Abstract])) OR (Intracranial Vascular Disease[Title/Abstract])) OR (Intracranial Vascular Diseases[Title/Abstract])) OR (Vascular Disease, Intracranial[Title/Abstract])) OR (Intracranial Vascular Disorders[Title/Abstract])) OR (Intracranial Vascular Disorder[Title/Abstract])) OR (Vascular Disorder, Intracranial[Title/Abstract])) OR (Vascular Disorders, Intracranial[Title/Abstract])) OR (Cerebrovascular Diseases[Title/Abstract])) OR (Cerebrovascular Disease[Title/Abstract])) OR (Disease, Cerebrovascular[Title/Abstract])) OR (Diseases, Cerebrovascular[Title/Abstract])) OR (Brain Vascular Disorders[Title/Abstract])) OR (Brain Vascular Disorder[Title/Abstract])) OR (Vascular Disorder, Brain[Title/Abstract])) OR (Vascular Disorders, Brain[Title/Abstract])) OR (Cerebrovascular Occlusion[Title/Abstract])) OR (Cerebrovascular Occlusions[Title/Abstract])) OR (Occlusion, Cerebrovascular[Title/Abstract])) OR (Occlusions, Cerebrovascular[Title/Abstract])) OR (Cerebrovascular Insufficiency[Title/Abstract])) OR (Cerebrovascular Insufficiencies[Title/Abstract])) OR (Insufficiencies, Cerebrovascular[Title/Abstract])) OR (Insufficiency, Cerebrovascular[Title/Abstract]) |
|  | #15 #9 OR #10 OR #11 OR #12 OR #13 OR #14 |
|  | #16 randomized controlled trial[Publication Type] |
|  | #17 #1 AND #8 AND #15 AND #16 |
|  | #18 #4 AND #8 AND #15 AND #16 |
| Embase | #1 'cerebrovascular disease'/exp OR 'basal ganglion hemorrhage'/exp OR 'brain hematoma'/exp OR 'brain hemorrhage'/exp OR 'brain infarction'/exp OR 'brain ischemia'/exp OR 'carotid artery disease'/exp OR 'cerebral artery disease'/exp OR 'cerebrovascular accident'/exp OR 'intracranial aneurysm'/exp OR 'occlusive cerebrovascular disease'/exp OR 'stroke patient'/exp OR 'stroke unit'/exp OR 'hemiparesis'/exp OR 'hemiplegia'/exp OR 'paresis'/exp OR 'neurologic gait disorder'/exp OR 'hemiplegic gait'/exp |
|  | #2 stroke:ti,ab,kw OR poststroke:ti,ab,kw OR 'post stroke':ti,ab,kw OR cerebrovasc*:ti,ab,kw OR 'brain vasc*':ti,ab,kw OR 'cerebral vasc*':ti,ab,kw OR apoplex*:ti,ab,kw OR sah:ti,ab,kw OR hemipleg*:ti,ab,kw OR hemipar*:ti,ab,kw OR paresis:ti,ab,kw OR paretic:ti,ab,kw OR TIA:ti,ab,kw OR ‘transient ischemic attack’:ti,ab,kw |
|  | #3 (brain*:ti,ab,kw OR cerebr*:ti,ab,kw OR cerebell*:ti,ab,kw OR intracran*:ti,ab,kw OR intracerebral:ti,ab,kw) AND (isch?emi*:ti,ab,kw OR infarct*:ti,ab,kw OR thrombo*:ti,ab,kw OR emboli*:ti,ab,kw OR occlus*:ti,ab,kw) |
|  | #4 (brain*:ti,ab,kw OR cerebr*:ti,ab,kw OR cerebell*:ti,ab,kw OR intracerebral:ti,ab,kw OR intracranial:ti,ab,kw OR subarachnoid:ti,ab,kw) AND (haemorrhage*:ti,ab,kw OR hemorrhage*:ti,ab,kw OR haematoma*:ti,ab,kw OR hematoma*:ti,ab,kw OR bleed*:ti,ab,kw) |
|  | #5 #1 OR #2 OR #3 OR #4 |
|  | #6 'exercise'/exp OR 'resistance training'/exp OR 'stretching exercise'/exp OR 'sport'/exp OR 'physical education'/exp OR 'dancing'/exp OR 'dance therapy'/exp OR 'weight bearing'/exp OR 'weight training'/exp OR 'walking'/exp OR 'body equilibrium'/exp OR 'motor activity'/exp OR 'physical activity'/exp OR 'yoga'/exp |
|  | #7 exercis*:ti,ab,kw OR exerciz*:ti,ab,kw OR sport:ti,ab,kw OR aerobic:ti,ab,kw OR 'physical fitness':ti,ab,kw OR 'weight bearing':ti,ab,kw OR 'load bearing':ti,ab,kw OR 'axial bearing':ti,ab,kw OR 'weight lifting':ti,ab,kw OR running:ti,ab,kw OR dancing:ti,ab,kw OR 'dance therapy':ti,ab,kw OR 'stair climb*':ti,ab,kw OR treadmill*:ti,ab,kw OR walk:ti,ab,kw OR walking:ti,ab,kw OR swim*:ti,ab,kw OR yoga:ti,ab,kw OR pilates:ti,ab,kw OR 'tai chi':ti,ab,kw OR 'tai ji':ti,ab,kw OR qigong:ti,ab,kw OR 'chi kung':ti,ab,kw OR gait:ti,ab,kw OR 'postural balance':ti,ab,kw OR 'body equilibrium':ti,ab,kw |
|  | #8 (resistance:ti,ab,kw OR strength:ti,ab,kw OR flexibility:ti,ab,kw OR balance:ti,ab,kw OR endurance:ti,ab,kw OR weight:ti,ab,kw OR agility:ti,ab,kw OR physical:ti,ab,kw OR circuit:ti,ab,kw OR motor:ti,ab,kw OR postural:ti,ab,kw) AND (train*:ti,ab,kw OR activit*:ti,ab,kw OR therapy:ti,ab,kw) |
|  | #9 #6 OR #7 OR #8 |
|  | #10 'randomized controlled trial':it OR random*:ti,ab,kw OR 'double blind':ti,ab,kw OR placebo:ti,ab,kw |
|  | #11 'anxiety'/exp OR 'anxiety disorder'/exp OR 'depression'/exp OR 'emotional disorder'/exp OR 'mental stress'/exp OR 'mental health'/exp OR 'quality of life'/exp |
|  | #12 anxi*:ti,ab,kw OR depress*:ti,ab,kw OR 'depressive disorder':ti,ab,kw OR 'quality of life':ti,ab,kw OR 'life quality':ti,ab,kw |
|  | #13 stress*:ti,ab,kw AND (psychological:ti,ab,kw OR emotional:ti,ab,kw) |
|  | #14 (mental:ti,ab,kw OR psychological:ti,ab,kw OR emotional:ti,ab,kw OR psychosocial:ti,ab,kw) AND (health:ti,ab,kw OR illness:ti,ab,kw OR wellbeing:ti,ab,kw) |
|  | #15 #11 OR #12 OR #13 OR #14 |
|  | #16 #5 AND #9 AND #10 AND #15 |
| Web of Science | #1 TS=( Cognition OR Cognitions OR Cognitive Function OR Cognitive Functions OR Function, Cognitive OR Functions, Cognitive ) |
|  | #2 (TS=(Anxiety OR Angst OR Social Anxiety OR Anxieties, Social OR Anxiety, Social OR Social Anxieties OR Hypervigilance OR Nervousness OR Anxiousness)) OR TS=(Depression OR Depressive Symptoms OR Depressive Symptom OR Symptom, Depressive OR Emotional Depression OR Depression, Emotional ) |
|  | #3 TS=(exercise OR Circuit-Based Exercise OR resistance training OR muscle stretching exercises OR sports OR physical education and training OR walking OR motor activity OR physical fitness OR exercis* OR exerciz* OR weight bearing OR dancing OR dance therapy OR stair climb* OR treadmill* OR walk OR walking OR swim* OR yoga OR pilates OR tai chi OR postural balance OR body equilibrium OR Exercise OR Exercises OR Physical Activity OR Activities, Physical OR Activity, Physical OR Physical Activities OR Exercise, Physical OR Exercises, Physical OR Physical Exercise OR Physical Exercises OR Acute Exercise OR Acute Exercises OR Exercise, Acute OR Exercises, Acute OR Exercise, Isometric OR Exercises, Isometric OR Isometric Exercises OR Isometric Exercise OR Exercise, Aerobic OR Aerobic Exercise OR Aerobic Exercises OR Exercises, Aerobic OR Exercise Training OR Exercise Trainings OR Training, Exercise OR Trainings, Exercise OR Sports OR Sport OR Athletics OR Athletic ) |
|  | #4 TS=( Stroke OR Strokes OR Cerebrovascular Accident OR Cerebrovascular Accidents OR CVA Cerebrovascular Accident OR CVAs Cerebrovascular Accident OR Cerebrovascular Apoplexy OR Apoplexy, Cerebrovascular OR Vascular Accident, Brain OR Brain Vascular Accident OR Brain Vascular Accidents OR Vascular Accidents, Brain OR Cerebrovascular Stroke OR Cerebrovascular Strokes OR Stroke, Cerebrovascular OR Strokes, Cerebrovascular OR Apoplexy OR Cerebral Stroke OR Cerebral Strokes OR Stroke, Cerebral OR Strokes, Cerebral OR Stroke, Acute OR Acute Stroke OR Acute Strokes OR Strokes, Acute OR Cerebrovascular Accident, Acute OR Acute Cerebrovascular Accident OR Acute Cerebrovascular Accidents OR Cerebrovascular Accidents, Acute OR Basal Ganglia Cerebrovascular Disease OR Vascular Diseases, Basal Ganglia OR Vascular Disease, Basal Ganglia OR Basal Ganglia Vascular Disease OR Cerebrovascular Disease, Basal Ganglia OR Lenticulostriate Vasculopathy OR Lenticulostriate Vasculopathies OR Vasculopathies, Lenticulostriate OR Vasculopathy, Lenticulostriate OR Lenticulostriate Vascular Diseases OR Lenticulostriate Vascular Disease OR Vascular Disease, Lenticulostriate OR Vascular Diseases, Lenticulostriate OR Lenticulostriate Diseases, Vascular OR Vascular Lenticulostriate Diseases OR Brain Infarction OR Brain Infarctions OR Infarction, Brain OR Infarctions, Brain OR Brain Infarct OR Brain Infarcts OR Infarct, Brain OR Infarcts, Brain OR Anterior Circulation Brain Infarction OR Infarction, Brain, Anterior Circulation OR Infarction, Anterior Circulation, Brain OR Anterior Circulation Infarction, Brain OR Brain Infarction, Anterior Circulation OR Venous Infarction, Brain OR Brain Venous Infarction OR Brain Venous Infarctions OR Infarction, Brain Venous OR Infarctions, Brain Venous OR Venous Infarctions, Brain OR Brain Infarction, Venous OR Brain Infarctions, Venous OR Infarction, Venous Brain OR Infarctions, Venous Brain OR Venous Brain Infarction OR Venous Brain Infarctions OR Anterior Cerebral Circulation Infarction OR Infarction, Anterior Cerebral Circulation OR Brain Infarction, Posterior Circulation OR Posterior Circulation Infarction, Brain OR Posterior Circulation Brain Infarction OR Infarction, Brain, Posterior Circulation OR Infarction, Posterior Circulation, Brain OR Paresis OR Pareses OR Muscular Paresis OR Muscular Pareses OR Pareses, Muscular OR Paresis, Muscular OR Muscle Paresis OR Muscle Pareses OR Pareses, Muscle OR Paresis, Muscle OR Monoparesis OR Monopareses OR Lower Extremity Paresis OR Extremity Pareses, Lower OR Extremity Paresis, Lower OR Lower Extremity Pareses OR Pareses, Lower Extremity OR Paresis, Lower Extremity OR Crural Paresis OR Crural Pareses OR Pareses, Crural OR Paresis, Crural OR Upper Extremity Paresis OR Extremity Pareses, Upper OR Extremity Paresis, Upper OR Pareses, Upper Extremity OR Paresis, Upper Extremity OR Upper Extremity Pareses OR Brachial Paresis OR Brachial Pareses OR Pareses, Brachial OR Paresis, Brachial OR Hemiparesis OR Hemipareses OR Paresis OR Pareses OR Muscular Paresis OR Muscular Pareses OR Pareses, Muscular OR Paresis, Muscular OR Muscle Paresis OR Muscle Pareses OR Pareses, Muscle OR Paresis, Muscle OR Monoparesis OR Monopareses OR Lower Extremity Paresis OR Extremity Pareses, Lower OR Extremity Paresis, Lower OR Lower Extremity Pareses OR Pareses, Lower Extremity OR Paresis, Lower Extremity OR Crural Paresis OR Crural Pareses OR Pareses, Crural OR Paresis, Crural OR Upper Extremity Paresis OR Extremity Pareses, Upper OR Extremity Paresis, Upper OR Pareses, Upper Extremity OR Paresis, Upper Extremity OR Upper Extremity Pareses OR Brachial Paresis OR Brachial Pareses OR Pareses, Brachial OR Paresis, Brachial OR Hemiparesis OR Hemipareses OR Hemiplegia OR Hemiplegias OR Hemiplegia, Transient OR Hemiplegias, Transient OR Transient Hemiplegia OR Transient Hemiplegias OR Monoplegia OR Monoplegias OR Hemiplegia, Post-Ictal OR Hemiplegia, Post Ictal OR Hemiplegias, Post-Ictal OR Post-Ictal Hemiplegia OR Post-Ictal Hemiplegias OR Hemiplegia, Crossed OR Crossed Hemiplegia OR Crossed Hemiplegias OR Hemiplegias, Crossed OR Hemiplegia, Flaccid OR Flaccid Hemiplegia OR Flaccid Hemiplegias OR Hemiplegias, Flaccid OR Hemiplegia, Infantile OR Hemiplegias, Infantile OR Infantile Hemiplegia OR Infantile Hemiplegias OR Hemiplegia, Spastic OR Hemiplegias, Spastic OR Spastic Hemiplegia OR Spastic Hemiplegias OR Cerebrovascular Disorders OR Cerebrovascular Disorder OR Vascular Diseases, Intracranial OR Intracranial Vascular Disease OR Intracranial Vascular Diseases OR Vascular Disease, Intracranial OR Intracranial Vascular Disorders OR Intracranial Vascular Disorder OR Vascular Disorder, Intracranial OR Vascular Disorders, Intracranial OR Cerebrovascular Diseases OR Cerebrovascular Disease OR Disease, Cerebrovascular OR Diseases, Cerebrovascular OR Brain Vascular Disorders OR Brain Vascular Disorder OR Vascular Disorder, Brain OR Vascular Disorders, Brain OR Cerebrovascular Occlusion OR Cerebrovascular Occlusions OR Occlusion, Cerebrovascular OR Occlusions, Cerebrovascular OR Cerebrovascular Insufficiency OR Cerebrovascular Insufficiencies OR Insufficiencies, Cerebrovascular OR Insufficiency, Cerebrovascular  ) |
|  | #5 TS=(randomized controlled trial OR random*) |
|  | #6 #2 AND #5 |
|  | #7 #1 AND #5 |
|  | #8 #3 AND #5 |
|  | #9 #4 AND #5 |
|  | #10 #9 AND #8 |
|  | #11 #6 AND #10 |
|  | #12 #7 AND #10 |
| Cochrane Library | #1 MeSH descriptor: [Depression] explode all trees |
|  | #2 MeSH descriptor: [Anxiety] explode all trees |
|  | #3 (Depressive Symptoms):ti,ab,kw OR (Depressive Symptom):ti,ab,kw OR (Symptom, Depressive):ti,ab,kw OR (Emotional Depression):ti,ab,kw OR (Depression, Emotional):ti,ab,kw (Word variations have been searched) in Trials |
|  | #4 MeSH descriptor: [Cognition] explode all trees |
|  | #5 (Angst OR Social Anxiety OR Anxieties, Social OR Anxiety, Social OR Social Anxieties OR Hypervigilance OR Nervousness OR Anxiousness):ti,ab,kw (Word variations have been searched) in Trials |
|  | #6 #1 OR #2 OR #3 OR #5 in Trials (Word variations have been searched) |
|  | #7 (Cognitions OR Cognitive Function OR Cognitive Functions OR Function, Cognitive OR Functions, Cognitive):ti,ab,kw (Word variations have been searched) in Trials |
|  | #8 #4 OR #7 in Trials (Word variations have been searched) |
|  | #9 MeSH descriptor: [Sports] explode all trees |
|  | #10 (Athletics OR Athletic OR Sport):ti,ab,kw |
|  | #11 MeSH descriptor: [Exercise] explode all trees |
|  | #12 (Exercises, Physical OR Physical Activities OR Exercise, Physical OR Activities, Physical OR Physical Exercises OR Activity, Physical OR Exercises OR Physical Activity OR Physical Exercise OR Acute Exercise OR Acute Exercises OR Exercise, Acute OR Exercises, Acute OR Training, Exercise OR Exercise Trainings OR Exercise Training OR Trainings, Exercise OR Exercises, Isometric OR Isometric Exercises OR Exercise, Isometric OR Isometric Exercise OR Aerobic Exercise OR Exercises, Aerobic OR Exercise, Aerobic OR Aerobic Exercises):ti,ab,kw (Word variations have been searched) in Trials |
|  | #13 #9 OR #10 OR #11 OR #12 in Trials (Word variations have been searched) |
|  | #14 #13 AND #6 in Trials (Word variations have been searched) |
|  | #15 #13 AND #8 in Trials (Word variations have been searched) |
|  | #16 MeSH descriptor: [Stroke] explode all trees |
|  | #17 MeSH descriptor: [Brain Infarction] explode all trees |
|  | #18 MeSH descriptor: [Paresis] explode all trees |
|  | #19 MeSH descriptor: [Hemiplegia] explode all trees |
|  | #20 MeSH descriptor: [Cerebrovascular Disorders] explode all trees |
|  | #21 (Cerebral Strokes OR Cerebral Stroke OR Vascular Accident, Brain OR Strokes OR Apoplexy, Cerebrovascular OR Apoplexy OR Cerebrovascular Apoplexy OR Cerebrovascular Stroke OR Stroke, Cerebrovascular OR Cerebrovascular Accident OR Brain Vascular Accident OR Cerebrovascular Accidents OR Brain Vascular Accidents OR Cerebrovascular Strokes OR Stroke, Cerebral OR CVAs (Cerebrovascular Accident) OR Strokes, Cerebrovascular OR CVA (Cerebrovascular Accident) OR Vascular Accidents, Brain OR Strokes, Cerebral OR Cerebrovascular Accidents, Acute OR Cerebrovascular Accident, Acute OR Acute Strokes OR Strokes, Acute OR Acute Stroke OR Acute Cerebrovascular Accident OR Acute Cerebrovascular Accidents OR Stroke, Acute):ti,ab,kw (Word variations have been searched) in Trials |
|  | #22 (Brain Venous Infarctions OR Venous Brain Infarctions OR Brain Infarctions, Venous OR Infarctions, Venous Brain OR Venous Infarctions, Brain OR Brain Venous Infarction OR Venous Brain Infarction OR Brain Infarction, Venous OR Infarction, Brain Venous OR Venous Infarction, Brain OR Infarctions, Brain Venous OR Infarction, Venous Brain OR Infarction, Anterior Cerebral Circulation OR Anterior Cerebral Circulation Infarction OR Anterior Circulation Infarction, Brain OR Brain Infarction, Anterior Circulation OR Infarction, Anterior Circulation, Brain OR Infarction, Brain, Anterior Circulation OR Anterior Circulation Brain Infarction OR Infarctions, Brain OR Brain Infarct OR Brain Infarcts OR Infarction, Brain OR Infarct, Brain OR Brain Infarctions OR Infarcts, Brain OR Infarction, Brain, Posterior Circulation OR Infarction, Posterior Circulation, Brain OR Posterior Circulation Infarction, Brain OR Posterior Circulation Brain Infarction OR Brain Infarction, Posterior Circulation):ti,ab,kw (Word variations have been searched) in Trials |
|  | #23 (Hemiparesis OR Hemipareses OR Brachial Pareses OR Upper Extremity Pareses OR Brachial Paresis OR Extremity Paresis, Upper OR Pareses, Brachial OR Pareses, Upper Extremity OR Paresis, Brachial OR Upper Extremity Paresis OR Extremity Pareses, Upper OR Paresis, Upper Extremity OR Muscle Paresis OR Muscular Pareses OR Pareses OR Muscular Paresis OR Pareses, Muscular OR Paresis, Muscle OR Muscle Pareses OR Pareses, Muscle OR Paresis, Muscular OR Lower Extremity Pareses OR Pareses, Crural OR Crural Paresis OR Pareses, Lower Extremity OR Crural Pareses OR Extremity Paresis, Lower OR Extremity Pareses, Lower OR Lower Extremity Paresis OR Paresis, Lower Extremity OR Paresis, Crural OR Monoparesis OR Monopareses):ti,ab,kw (Word variations have been searched) in Trials |
|  | #24 (Hemiplegias, Crossed OR Hemiplegia, Crossed OR Crossed Hemiplegia OR Crossed Hemiplegias OR Monoplegias OR Monoplegia OR Hemiplegias, Transient OR Transient Hemiplegia OR Transient Hemiplegias OR Hemiplegia, Transient OR Infantile Hemiplegia OR Infantile Hemiplegias OR Hemiplegia, Infantile OR Hemiplegias, Infantile OR Flaccid Hemiplegia OR Flaccid Hemiplegias OR Hemiplegias, Flaccid OR Hemiplegia, Flaccid OR Hemiplegias OR Hemiplegia, Spastic OR Hemiplegias, Spastic OR Spastic Hemiplegia OR Spastic Hemiplegias OR Hemiplegias, Post-Ictal OR Hemiplegia, Post Ictal OR Hemiplegia, Post-Ictal OR Post-Ictal Hemiplegia OR Post-Ictal Hemiplegias):ti,ab,kw (Word variations have been searched) in Trials |
|  | #25 (Cerebrovascular Insufficiency OR Cerebrovascular Insufficiencies OR Insufficiencies, Cerebrovascular OR Insufficiency, Cerebrovascular OR Cerebrovascular Occlusions OR Cerebrovascular Occlusion OR Occlusion, Cerebrovascular OR Occlusions, Cerebrovascular OR Cerebrovascular Disorder OR Cerebrovascular Disease OR Intracranial Vascular Disorder OR Intracranial Vascular Disorders OR Brain Vascular Disorders OR Vascular Diseases, Intracranial OR Cerebrovascular Diseases OR Vascular Disorder, Brain OR Diseases, Cerebrovascular OR Vascular Disorders, Brain OR Intracranial Vascular Disease OR Vascular Disorders, Intracranial OR Brain Vascular Disorder OR Disease, Cerebrovascular OR Intracranial Vascular Diseases OR Vascular Disorder, Intracranial OR Vascular Disease, Intracranial):ti,ab,kw (Word variations have been searched) in Trials |
|  | #26 #21 OR #22 OR #23 OR #24 OR #25 in Trials (Word variations have been searched) |
|  | #27 #26 AND #14 in Trials (Word variations have been searched) |
|  | #28 #26 AND #15 in Trials (Word variations have been searched) |
| CNKI | #1 SU=运动 OR SU=有氧运动 OR SU=体育锻炼 OR SU=运动训练 OR SU=有氧训练 OR SU=运动锻炼 OR SU=八段绵 OR SU=气功 OR SU=太极拳法 OR SU=舞蹈 OR SU=瑜伽 |
|  | #2 SU=脑卒中 OR SU=中风 OR SU=脑血管障碍 OR SU=脑梗死 OR SU=脑栓塞 OR SU=脑出血 OR SU=颅内出血 OR SU=脑卒中后 OR SU=脑血管病 |
|  | #3 SU=抑郁 OR SU=抑郁症 OR SU=抑郁障碍 OR SU=抑郁症状 OR SU=焦虑症 OR SU=焦虑 OR SU=焦虑障碍 OR SU=焦虑症状 |
|  | #4 SU=随机对照实验 OR SU=随机 OR SU=临床观察 OR SU=疗效 |
|  | #5 (SU=运动 OR SU=有氧运动 OR SU=体育锻炼 OR SU=运动训练 OR SU=有氧训练 OR SU=运动锻炼 OR SU=八段绵 OR SU=气功 OR SU=太极拳法 OR SU=舞蹈 OR SU=瑜伽) AND(SU=脑卒中 OR SU=中风 OR SU=脑血管障碍 OR SU=脑梗死 OR SU=脑栓塞 OR SU=脑出血 OR SU=颅内出血 OR SU=脑卒中后 OR SU=脑血管病) AND (SU=抑郁 OR SU=抑郁症 OR SU=抑郁障碍 OR SU=抑郁症状 OR SU=焦虑症 OR SU=焦虑 OR SU=焦虑障碍 OR SU=焦虑症状)AND (SU=随机对照实验 OR SU=随机 OR SU=临床观察 OR SU=疗效) |
|  | #6 (SU=运动 OR SU=有氧运动 OR SU=体育锻炼 OR SU=运动训练 OR SU=有氧训练 OR SU=运动锻炼 OR SU=八段绵 OR SU=气功 OR SU=太极拳法 OR SU=舞蹈 OR SU=瑜伽) AND(SU=脑卒中 OR SU=中风 OR SU=脑血管障碍 OR SU=脑梗死 OR SU=脑栓塞 OR SU=脑出血 OR SU=颅内出血 OR SU=脑卒中后 OR SU=脑血管病) AND(SU=认知障碍 OR SU=记忆障碍 OR SU=认知功能障碍 OR SU=认知功能损害 OR SU=阿尔茨海默病) AND (SU=随机对照实验 OR SU=随机 OR SU=临床观察 OR SU=疗效) |
| VIPC | #1 M=脑卒中OR M=中风OR M=脑血管障碍OR M=脑梗死OR M=脑栓塞OR M=脑出血OR M=颅内出血OR M=[脑卒中后](https://kns.cnki.net/kcms2/keyword/detail?v=0qMDjMp0v1ka5kYucogEeDDy3-hTGHbPMsBr3Z5gtmht4zBc4vM4CAD6JzfuzKxP3ZBGTLHzmeeFGEyXlASSVj2xoYE9vqhDgsgKoSysN6X4-qsgtE38Aw==&uniplatform=NZKPT&language=CHS)OR M=脑血管病 |
|  | #2 M=抑郁OR M=抑郁症OR M=抑郁障碍OR M=[抑郁症状](https://kns.cnki.net/kcms2/keyword/detail?v=0qMDjMp0v1l7jREoAUSJdStThQcKbPK7EtpxaBBluCwUCcKcCOwPXh-GUK4GqyILX7miSdJwml8N5-hAtgaLY2ASyCZ0bx56NoeIH8PAo8OytKmApUd1Yw==&uniplatform=NZKPT&language=CHS)OR M=焦虑症OR M=焦虑OR M=焦虑障碍OR M=[焦虑症状](https://kns.cnki.net/kcms2/keyword/detail?v=0qMDjMp0v1l7jREoAUSJdStThQcKbPK7EtpxaBBluCwUCcKcCOwPXh-GUK4GqyILX7miSdJwml8N5-hAtgaLY2ASyCZ0bx56NoeIH8PAo8OytKmApUd1Yw==&uniplatform=NZKPT&language=CHS) |
|  | #3 M=认知障碍OR M=[记忆障碍](https://kns.cnki.net/kcms2/keyword/detail?v=0qMDjMp0v1lGxA5U2v5w9-Ri7ZopVQfel4bI3X_lPBSZHOyJsy6JY76s1kQcaET0NEVdU4mC_VRvCRs36ynuNrvtqQwgWrgkKad-rIZTisnvIN3MRuQw-g==&uniplatform=NZKPT&language=CHS)OR M=[认知功能障碍](https://kns.cnki.net/kcms2/keyword/detail?v=0qMDjMp0v1lGxA5U2v5w9-Ri7ZopVQfel4bI3X_lPBSZHOyJsy6JY76s1kQcaET0NEVdU4mC_VRvCRs36ynuNrvtqQwgWrgkKad-rIZTisnvIN3MRuQw-g==&uniplatform=NZKPT&language=CHS)OR M=[认知功能损害](https://kns.cnki.net/kcms2/keyword/detail?v=0qMDjMp0v1lGxA5U2v5w9-Ri7ZopVQfep_GyeciOrTFldhU-z0-1XHJX7W31TmxkIDVzJGA-ACMtk3mz8qQaNhbVKvdMlHjHD9N7Q6mdpp2eopjFaFTuGG_uTKUDZLshPT0t5EhFe0k=&uniplatform=NZKPT&language=CHS)OR M=[阿尔茨海默病](https://kns.cnki.net/kcms2/keyword/detail?v=0qMDjMp0v1lGxA5U2v5w9-Ri7ZopVQfeVLIHNCAN0AjiD_SPLF_8N2zGgNxFCt1IK6IhsZiV_cjmyuTmF8G6gZeo-zexLLioy6QAD92nIS7pcvIjC7op_EoSQDqtcuVXgrYSSlpyYWw=&uniplatform=NZKPT&language=CHS) |
|  | #4 M=运动OR M=有氧运动OR M=体育锻炼OR M=运动训练OR M=有氧训练OR M=运动锻炼OR M=[八段绵](https://kns.cnki.net/kcms2/keyword/detail?v=0qMDjMp0v1mxkwAgm66dvZGyE3FxjD8Q2uNAxsG9CHR1zuiuvTScZUA6db9p0-CvYLjfVhTdJcQwNU8y_Ypczwia9SvWp-SVC_qRWywVzwI=&uniplatform=NZKPT&language=CHS)OR M=气功OR M=太极拳法OR M=舞蹈OR M=瑜伽 |
|  | #5 M=随机对照试验OR M=随机OR M=临床观察OR M=疗效 |
|  | #6 (M=脑卒中 OR M=中风 OR M=脑血管障碍 OR M=脑梗死 OR M=脑栓塞 OR M=脑出血 OR M=颅内出血 OR M=[脑卒中后](https://kns.cnki.net/kcms2/keyword/detail?v=0qMDjMp0v1ka5kYucogEeDDy3-hTGHbPMsBr3Z5gtmht4zBc4vM4CAD6JzfuzKxP3ZBGTLHzmeeFGEyXlASSVj2xoYE9vqhDgsgKoSysN6X4-qsgtE38Aw==&uniplatform=NZKPT&language=CHS) OR M=脑血管病) AND (M=抑郁 OR M=抑郁症 OR M=抑郁障碍 OR M=[抑郁症状](https://kns.cnki.net/kcms2/keyword/detail?v=0qMDjMp0v1l7jREoAUSJdStThQcKbPK7EtpxaBBluCwUCcKcCOwPXh-GUK4GqyILX7miSdJwml8N5-hAtgaLY2ASyCZ0bx56NoeIH8PAo8OytKmApUd1Yw==&uniplatform=NZKPT&language=CHS) OR M=焦虑症 OR M=焦虑 OR M=焦虑障碍 OR M=[焦虑症状](https://kns.cnki.net/kcms2/keyword/detail?v=0qMDjMp0v1l7jREoAUSJdStThQcKbPK7EtpxaBBluCwUCcKcCOwPXh-GUK4GqyILX7miSdJwml8N5-hAtgaLY2ASyCZ0bx56NoeIH8PAo8OytKmApUd1Yw==&uniplatform=NZKPT&language=CHS)) AND (M=运动 OR M=有氧运动 OR M=体育锻炼 OR M=运动训练 OR M=有氧训练 OR M=运动锻炼 OR M=[八段绵](https://kns.cnki.net/kcms2/keyword/detail?v=0qMDjMp0v1mxkwAgm66dvZGyE3FxjD8Q2uNAxsG9CHR1zuiuvTScZUA6db9p0-CvYLjfVhTdJcQwNU8y_Ypczwia9SvWp-SVC_qRWywVzwI=&uniplatform=NZKPT&language=CHS) OR M=气功 OR M=太极拳法 OR M=舞蹈 OR M=瑜伽) AND (M=随机对照试验 OR M=随机 OR M=临床观察 OR M=疗效) |
|  | #7 (M=脑卒中 OR M=中风 OR M=脑血管障碍 OR M=脑梗死 OR M=脑栓塞 OR M=脑出血 OR M=颅内出血 OR M=[脑卒中后](https://kns.cnki.net/kcms2/keyword/detail?v=0qMDjMp0v1ka5kYucogEeDDy3-hTGHbPMsBr3Z5gtmht4zBc4vM4CAD6JzfuzKxP3ZBGTLHzmeeFGEyXlASSVj2xoYE9vqhDgsgKoSysN6X4-qsgtE38Aw==&uniplatform=NZKPT&language=CHS) OR M=脑血管病) AND (M=认知障碍 OR M=[记忆障碍](https://kns.cnki.net/kcms2/keyword/detail?v=0qMDjMp0v1lGxA5U2v5w9-Ri7ZopVQfel4bI3X_lPBSZHOyJsy6JY76s1kQcaET0NEVdU4mC_VRvCRs36ynuNrvtqQwgWrgkKad-rIZTisnvIN3MRuQw-g==&uniplatform=NZKPT&language=CHS) OR M=[认知功能障碍](https://kns.cnki.net/kcms2/keyword/detail?v=0qMDjMp0v1lGxA5U2v5w9-Ri7ZopVQfel4bI3X_lPBSZHOyJsy6JY76s1kQcaET0NEVdU4mC_VRvCRs36ynuNrvtqQwgWrgkKad-rIZTisnvIN3MRuQw-g==&uniplatform=NZKPT&language=CHS) OR M=[认知功能损害](https://kns.cnki.net/kcms2/keyword/detail?v=0qMDjMp0v1lGxA5U2v5w9-Ri7ZopVQfep_GyeciOrTFldhU-z0-1XHJX7W31TmxkIDVzJGA-ACMtk3mz8qQaNhbVKvdMlHjHD9N7Q6mdpp2eopjFaFTuGG_uTKUDZLshPT0t5EhFe0k=&uniplatform=NZKPT&language=CHS) OR M=[阿尔茨海默病](https://kns.cnki.net/kcms2/keyword/detail?v=0qMDjMp0v1lGxA5U2v5w9-Ri7ZopVQfeVLIHNCAN0AjiD_SPLF_8N2zGgNxFCt1IK6IhsZiV_cjmyuTmF8G6gZeo-zexLLioy6QAD92nIS7pcvIjC7op_EoSQDqtcuVXgrYSSlpyYWw=&uniplatform=NZKPT&language=CHS)) AND (M=运动 OR M=有氧运动 OR M=体育锻炼 OR M=运动训练 OR M=有氧训练 OR M=运动锻炼 OR M=[八段绵](https://kns.cnki.net/kcms2/keyword/detail?v=0qMDjMp0v1mxkwAgm66dvZGyE3FxjD8Q2uNAxsG9CHR1zuiuvTScZUA6db9p0-CvYLjfVhTdJcQwNU8y_Ypczwia9SvWp-SVC_qRWywVzwI=&uniplatform=NZKPT&language=CHS) OR M=气功 OR M=太极拳法 OR M=舞蹈 OR M=瑜伽) AND (M=随机对照试验 OR M=随机 OR M=临床观察 OR M=疗效) |
| Wanfang Data | (主题:(脑卒中) or 主题:(中风) or 主题:(脑血管障碍) or 主题:(脑梗死) or 主题:(脑栓塞)or 主题:(脑出血) or 主题:(颅内出血) or 主题:([脑卒中后](https://kns.cnki.net/kcms2/keyword/detail?v=0qMDjMp0v1ka5kYucogEeDDy3-hTGHbPMsBr3Z5gtmht4zBc4vM4CAD6JzfuzKxP3ZBGTLHzmeeFGEyXlASSVj2xoYE9vqhDgsgKoSysN6X4-qsgtE38Aw==&uniplatform=NZKPT&language=CHS)) or 主题:(脑血管病） |
|  | (主题:(认知障碍) or 主题:([记忆障碍](https://kns.cnki.net/kcms2/keyword/detail?v=0qMDjMp0v1lGxA5U2v5w9-Ri7ZopVQfel4bI3X_lPBSZHOyJsy6JY76s1kQcaET0NEVdU4mC_VRvCRs36ynuNrvtqQwgWrgkKad-rIZTisnvIN3MRuQw-g==&uniplatform=NZKPT&language=CHS)) or 主题:([认知功能障碍](https://kns.cnki.net/kcms2/keyword/detail?v=0qMDjMp0v1lGxA5U2v5w9-Ri7ZopVQfel4bI3X_lPBSZHOyJsy6JY76s1kQcaET0NEVdU4mC_VRvCRs36ynuNrvtqQwgWrgkKad-rIZTisnvIN3MRuQw-g==&uniplatform=NZKPT&language=CHS)) or 主题:([认知功能障碍](https://kns.cnki.net/kcms2/keyword/detail?v=0qMDjMp0v1lGxA5U2v5w9-Ri7ZopVQfel4bI3X_lPBSZHOyJsy6JY76s1kQcaET0NEVdU4mC_VRvCRs36ynuNrvtqQwgWrgkKad-rIZTisnvIN3MRuQw-g==&uniplatform=NZKPT&language=CHS)) or 主题:([阿尔茨海默病](https://kns.cnki.net/kcms2/keyword/detail?v=0qMDjMp0v1lGxA5U2v5w9-Ri7ZopVQfeVLIHNCAN0AjiD_SPLF_8N2zGgNxFCt1IK6IhsZiV_cjmyuTmF8G6gZeo-zexLLioy6QAD92nIS7pcvIjC7op_EoSQDqtcuVXgrYSSlpyYWw=&uniplatform=NZKPT&language=CHS)) |
|  | (主题:(抑郁) or 主题:(抑郁症) or 主题:(抑郁障碍) or 主题:([抑郁症状](https://kns.cnki.net/kcms2/keyword/detail?v=0qMDjMp0v1l7jREoAUSJdStThQcKbPK7EtpxaBBluCwUCcKcCOwPXh-GUK4GqyILX7miSdJwml8N5-hAtgaLY2ASyCZ0bx56NoeIH8PAo8OytKmApUd1Yw==&uniplatform=NZKPT&language=CHS)) or 主题:(焦虑症) or 主题:(焦虑) or 主题:(焦虑障碍) or 主题:([焦虑症状](https://kns.cnki.net/kcms2/keyword/detail?v=0qMDjMp0v1l7jREoAUSJdStThQcKbPK7EtpxaBBluCwUCcKcCOwPXh-GUK4GqyILX7miSdJwml8N5-hAtgaLY2ASyCZ0bx56NoeIH8PAo8OytKmApUd1Yw==&uniplatform=NZKPT&language=CHS)) |
|  | (主题:(运动) or 主题:(有氧运动) or 主题:(体育锻炼) or 主题:(运动训练) or 主题:(有氧训练) or 主题:(运动锻炼) or 主题:([八段绵](https://kns.cnki.net/kcms2/keyword/detail?v=0qMDjMp0v1mxkwAgm66dvZGyE3FxjD8Q2uNAxsG9CHR1zuiuvTScZUA6db9p0-CvYLjfVhTdJcQwNU8y_Ypczwia9SvWp-SVC_qRWywVzwI=&uniplatform=NZKPT&language=CHS)) or 主题:(气功) or 主题:(太极拳法) or 主题:(舞蹈) or 主题:(瑜伽) |
|  | (主题:(随机对照试验) or 主题:(随机) or 主题:(临床观察) or 主题:(疗效) |
|  | (主题:(脑卒中) or 主题:(中风) or 主题:(脑血管障碍) or 主题:(脑梗死) or 主题:(脑栓塞)or 主题:(脑出血) or 主题:(颅内出血) or 主题:([脑卒中后](https://kns.cnki.net/kcms2/keyword/detail?v=0qMDjMp0v1ka5kYucogEeDDy3-hTGHbPMsBr3Z5gtmht4zBc4vM4CAD6JzfuzKxP3ZBGTLHzmeeFGEyXlASSVj2xoYE9vqhDgsgKoSysN6X4-qsgtE38Aw==&uniplatform=NZKPT&language=CHS)) or 主题:(脑血管病) AND (主题:(认知障碍) or 主题:([记忆障碍](https://kns.cnki.net/kcms2/keyword/detail?v=0qMDjMp0v1lGxA5U2v5w9-Ri7ZopVQfel4bI3X_lPBSZHOyJsy6JY76s1kQcaET0NEVdU4mC_VRvCRs36ynuNrvtqQwgWrgkKad-rIZTisnvIN3MRuQw-g==&uniplatform=NZKPT&language=CHS)) or 主题:([认知功能障碍](https://kns.cnki.net/kcms2/keyword/detail?v=0qMDjMp0v1lGxA5U2v5w9-Ri7ZopVQfel4bI3X_lPBSZHOyJsy6JY76s1kQcaET0NEVdU4mC_VRvCRs36ynuNrvtqQwgWrgkKad-rIZTisnvIN3MRuQw-g==&uniplatform=NZKPT&language=CHS)) or 主题:([认知功能障碍](https://kns.cnki.net/kcms2/keyword/detail?v=0qMDjMp0v1lGxA5U2v5w9-Ri7ZopVQfel4bI3X_lPBSZHOyJsy6JY76s1kQcaET0NEVdU4mC_VRvCRs36ynuNrvtqQwgWrgkKad-rIZTisnvIN3MRuQw-g==&uniplatform=NZKPT&language=CHS)) or 主题:([阿尔茨海默病](https://kns.cnki.net/kcms2/keyword/detail?v=0qMDjMp0v1lGxA5U2v5w9-Ri7ZopVQfeVLIHNCAN0AjiD_SPLF_8N2zGgNxFCt1IK6IhsZiV_cjmyuTmF8G6gZeo-zexLLioy6QAD92nIS7pcvIjC7op_EoSQDqtcuVXgrYSSlpyYWw=&uniplatform=NZKPT&language=CHS)) AND (主题:(运动) or 主题:(有氧运动) or 主题:(体育锻炼) or 主题:(运动训练) or 主题:(有氧训练) or 主题:(运动锻炼) or 主题:([八段绵](https://kns.cnki.net/kcms2/keyword/detail?v=0qMDjMp0v1mxkwAgm66dvZGyE3FxjD8Q2uNAxsG9CHR1zuiuvTScZUA6db9p0-CvYLjfVhTdJcQwNU8y_Ypczwia9SvWp-SVC_qRWywVzwI=&uniplatform=NZKPT&language=CHS)) or 主题:(气功) or 主题:(太极拳法) or 主题:(舞蹈) or 主题:(瑜伽) AND (主题:(随机对照试验) or 主题:(随机) or 主题:(临床观察) or 主题:(疗效) |
|  | (主题:(脑卒中) or 主题:(中风) or 主题:(脑血管障碍) or 主题:(脑梗死) or 主题:(脑栓塞)or 主题:(脑出血) or 主题:(颅内出血) or 主题:([脑卒中后](https://kns.cnki.net/kcms2/keyword/detail?v=0qMDjMp0v1ka5kYucogEeDDy3-hTGHbPMsBr3Z5gtmht4zBc4vM4CAD6JzfuzKxP3ZBGTLHzmeeFGEyXlASSVj2xoYE9vqhDgsgKoSysN6X4-qsgtE38Aw==&uniplatform=NZKPT&language=CHS)) or 主题:(脑血管病) AND(主题:(抑郁) or 主题:(抑郁症) or 主题:(抑郁障碍) or 主题:([抑郁症状](https://kns.cnki.net/kcms2/keyword/detail?v=0qMDjMp0v1l7jREoAUSJdStThQcKbPK7EtpxaBBluCwUCcKcCOwPXh-GUK4GqyILX7miSdJwml8N5-hAtgaLY2ASyCZ0bx56NoeIH8PAo8OytKmApUd1Yw==&uniplatform=NZKPT&language=CHS)) or 主题:(焦虑症) or 主题:(焦虑) or 主题:(焦虑障碍) or 主题:([焦虑症状](https://kns.cnki.net/kcms2/keyword/detail?v=0qMDjMp0v1l7jREoAUSJdStThQcKbPK7EtpxaBBluCwUCcKcCOwPXh-GUK4GqyILX7miSdJwml8N5-hAtgaLY2ASyCZ0bx56NoeIH8PAo8OytKmApUd1Yw==&uniplatform=NZKPT&language=CHS)) AND (主题:(运动) or 主题:(有氧运动) or 主题:(体育锻炼) or 主题:(运动训练) or 主题:(有氧训练) or 主题:(运动锻炼) or 主题:([八段绵](https://kns.cnki.net/kcms2/keyword/detail?v=0qMDjMp0v1mxkwAgm66dvZGyE3FxjD8Q2uNAxsG9CHR1zuiuvTScZUA6db9p0-CvYLjfVhTdJcQwNU8y_Ypczwia9SvWp-SVC_qRWywVzwI=&uniplatform=NZKPT&language=CHS)) or 主题:(气功) or 主题:(太极拳法) or 主题:(舞蹈) or 主题:(瑜伽) AND (主题:(随机对照试验) or 主题:(随机) or 主题:(临床观察) or 主题:(疗效) |
